# Cryo-EM structures of the human NaS1 and NaDC1 transporters revealed the elevator transport and allosteric regulation mechanism

**DOI:** 10.1126/sciadv.adl3685

**Published:** 2024-03-29

**Authors:** Ximin Chi, Yiming Chen, Yaning Li, Lu Dai, Yuanyuan Zhang, Yaping Shen, Yun Chen, Tianhao Shi, Haonan Yang, Zilong Wang, Renhong Yan

**Affiliations:** ^1^ State Key Laboratory of Cellular Stress Biology, Innovation Center for Cell Signaling Network, School of Life Science, Xiamen University, Xiamen 361102, Fujian Province, China.; ^2^ Center for Infectious Disease Research, Westlake Laboratory of Life Sciences and Biomedicine, Key Laboratory of Structural Biology of Zhejiang Province, School of Life Sciences, Westlake University, Hangzhou 310024, Zhejiang Province, China.; ^3^ Department of Medical Neuroscience, Key University Laboratory of Metabolism and Health of Guangdong, School of Medicine, Southern University of Science and Technology, Shenzhen 518055, Guangdong Province, China.; ^4^ Department of Biochemistry, Key University Laboratory of Metabolism and Health of Guangdong, School of Medicine, Institute for Biological Electron Microscopy, Southern University of Science and Technology, Shenzhen 518055, Guangdong Province, China.; ^5^ Beijing Advanced Innovation Center for Structural Biology, Tsinghua-Peking Joint Center for Life Sciences, School of Life Sciences, Tsinghua University, Beijing 100084, China.; ^6^ Novoprotein Scientific Inc., Suzhou 215000, China.

## Abstract

The solute carrier 13 (SLC13) family comprises electrogenic sodium ion–coupled anion cotransporters, segregating into sodium ion–sulfate cotransporters (NaSs) and sodium ion–di- and–tricarboxylate cotransporters (NaDCs). NaS1 and NaDC1 regulate sulfate homeostasis and oxidative metabolism, respectively. NaS1 deficiency affects murine growth and fertility, while NaDC1 affects urinary citrate and calcium nephrolithiasis. Despite their importance, the mechanisms of substrate recognition and transport remain insufficiently characterized. In this study, we determined the cryo–electron microscopy structures of human NaS1, capturing inward-facing and combined inward-facing/outward-facing conformations within a dimer both in apo and sulfate-bound states. In addition, we elucidated NaDC1’s outward-facing conformation, encompassing apo, citrate-bound, and *N*-(*p*-amylcinnamoyl) anthranilic acid (ACA) inhibitor–bound states. Structural scrutiny illuminates a detailed elevator mechanism driving conformational changes. Notably, the ACA inhibitor unexpectedly binds primarily anchored by transmembrane 2 (TM2), Loop 10, TM11, and TM6a proximate to the cytosolic membrane. Our findings provide crucial insights into SLC13 transport mechanisms, paving the way for future drug design.

## Teaser

The multiple working states of SLC13 family members reveal the detailed transport mechanism.

## INTRODUCTION

The solute carrier 13 (SLC13) family consists of two distinct groups of closely related proteins: Na^+^-sulfate cotransporters (NaSs) and Na^+^-di- and tricarboxylate cotransporters (NaDCs) (*1*–*3*). NaS cotransporters, represented by NaS1 (SLC13A1) and NaS2 (SLC13A4), primarily facilitate the transport of sulfate, selenate, and thiosulfate (*4*, *5*). In contrast, NaDCs, including NaDC1 (SLC13A2), NaDC3 (SLC13A3), and NaCT (sodium-coupled citrate transporter, SLC13A5), orchestrate the movement of Krebs cycle intermediates such as succinate, citrate, and α-ketoglutarate (*6*–*8*). These members of the SLC13 family display cross-species conservation and play crucial roles in sulfate homeostasis and oxidative metabolism, making them attractive targets for drug development under conditions such as hypertension, nephrolithiasis, and epilepsy (*9*–*12*).

NaDC1, a representative member of SLC13 family, mediates both pH-independent, sodium-dependent cotransport of succinate and pH- and sodium-dependent cotransport of citrate within the kidney and small intestine (*6*, *13*, *14*). Despite sharing over 45% amino acid identity among carboxylate transporters, such as NaDC1, NaDC3, and NaCT, their distinct expression and distribution patterns set them apart (fig. S1) (*15*). The citrate reabsorption of NaDC1 is critical for regulating the level of urinary citrate in the renal proximal tubule, whose abnormality is closely related to calcium nephrolithiasis (*12*, *16*). The inactivation of NaDC1 homologs found in *Drosophila melanogaster* and *Caenorhabditis elegans* could increase the lifespan notably, underscoring the importance of targeting NaDC1 for aging-related research (*17*–*19*). It has been reported that *N*-(*p*-amylcinnamoyl) anthranilic acid (ACA) and 2-(*p*-amylcinnamoyl)amino-4-chlorobenzoic acid (ONO-RS-082) could selectively inhibit the transport activity of human NaDC1, rather than the NaS1 (*20*). However, the precise mechanism of this inhibition remains to be fully elucidated.

Conversely, NaS1 primarily participates in pH-independent, sodium-dependent cotransport of sulfate across renal proximal tubules and small intestinal epithelia (*21*–*23*). NaS1 is crucial for maintaining the sulfate homeostasis in the human body (*24*). Sulfate ions are essential for various biological processes, including the synthesis of sulfur-containing compounds, such as proteins, and the formation of sulfate esters in glycosaminoglycans (*1*, *25*). The *SLC13A1* knockout mouse models show notable hyposulfatemia and hypersulfaturia, suggesting the essential role of NaS1 in maintaining sulfate homeostasis of which perturbations could influence a wide range of physiological processes including metabolism, growth, behavior, and fecundity (*26*–*29*).

Extensive structural investigation into the bacterial homologs of the SLC13 family has unveiled an 11–transmembrane domain (TMD) topology that forms a dimeric state, along with an elevator-type transport mechanism (*30*–*33*). Notably, Sauer *et al.* (*34*) reported the first structure of human sodium-dependent citrate transporter NaCT and revealed the substrate and inhibitor bound inward facing conformation, providing an important clue to optimize the anti-obesity drugs. However, comprehensive architectural insights into other human SLC13 members remain scarce, which, in turn, limits our understanding of substrate selectivity and inhibition mechanisms. In light of this, we present our cryo–electron microscopy (cryo-EM)–derived structures of human NaS1 and NaDC1, capturing multiple conformations during transport cycle. Through these structures, we systematically unveil the intricacies of substrate recognition and conformational changes within human SLC13 members.

## RESULTS

### Structural determination of human NaS1 and NaDC1

**Fig. 1.**
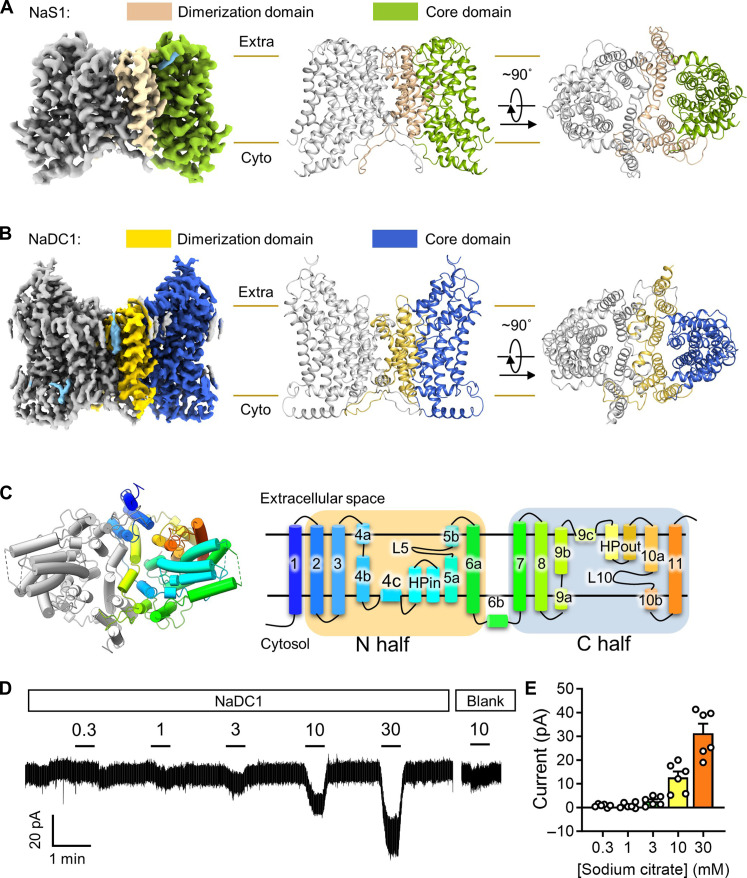
Overall structures of human NaDC1 and NaS1. (**A**) Cryo-EM map and overall structure of NaS1. Scaffold (or Dimerization) and core domain of NaS1 are colored in wheat and green, respectively. (**B**) Cryo-EM map and overall structure of NaDC1. Scaffold (or Dimerization) and core domain of NaDC1 are colored in yellow and blue, respectively. (**C**) Overall structure of human NaDC1. Left: Molecular model of NaDC1. One protomer is rainbow-colored, and the other is colored gray. Right: Scheme of one NaDC1 protomer with the same coloring pattern. (**D** and **E**) Representative trace and quantification of currents induced by gradient concentration of citrate (in millimolars) on NaDC1 transfected or blank HEK293T cells. Data present means ± SEM.

To delve deeper into the transport mechanism of human SLC13 members, we used single-particle cryo-EM technique to reveal intricate structural insights. For human NaS1, we captured both inward-facing conformations, namely, apo (NaS1_apo_IN/IN) and sulfate-bound states (NaS1_sulfate_IN/IN), achieving resolutions of 2.9 and 3.1 Å, respectively. In addition, we successfully elucidated the simultaneous presence of inward-facing and outward-facing conformations within a dimer, both in apo (NaS1_apo_IN/OUT) and sulfate-bound states (NaS1_sulfate_IN/OUT), at resolutions of 3 and 3.2 Å, respectively (Fig. 1A, table S1, and figs. S2A and S3 to S6, S9). Furthermore, we achieved cryo-EM visualization of NaDC1 across distinct states: apo (NaDC1_apo), citrate bound (NaDC1_citrate), and ACA bound (NaDC1_ACA), all exhibiting outward-facing conformations, with resolutions of 2.5, 2.7, and 3.3 Å, respectively (Fig. 1, B and C; table S2; and figs. S2B, S7 to S9). For clarity, we have designated the NaS1 conformations as “IN/IN” for the inward-facing state, “IN/OUT” for the combined inward-facing/outward-facing states within a dimer, and “OUT/OUT” for the outward-facing conformation of NaDC1. Further details regarding protein purification, sample preparation, data processing, and model construction can be found in Materials and Methods. In this section, we will predominantly focus on the detailed structural analysis of the apo structure of NaDC1.

From an overall view, both the NaDC1 and NaS1 form a homodimer, and each protomer consists of 11 TMDs (Fig. 1, A to C). The dimeric interface is mediated by the scaffold domain that includes TM1 to TM4 and TM7 to TM9 (which is also named as dimerization domain). The rest TMDs form the core domain that is responsible for substrate recognition and conformational change to complete the transport cycle (Fig. 1, A and B). The architecture supports the elevator transport model of this protein family (*30*, *32*, *34*). TM2 to TM6 form the N half of the protein, while TM7 to TM11 form the C half (Fig. 1C). Both halves are arranged inversely in the bilayer membrane. Notably, the intracellular loop (residue 165 to 212 amino acids) of NaDC1 that connects the HPin and TM5 was poorly resolved because of its flexibility. In addition, the rest sequences of NaDC1 were clearly resolved, especially the preTM1 region (residue 1 to 12 amino acids) and intracellular loop 8 to 9 (IL_8–9_; residue 383 to 409 amino acids), of which the counterpart has not been resolved in human NaCT structure (*34*). Among them, TM4, TM5, TM6, TM9, and TM10 are “broken” to several α helices within the membrane region. The two hairpin segments (HPin and HPout), loop 5 (L5) between TM5a and TM5b, and L10 between TM10a and TM10b are essential for the coordination of substrate and sodium ions (Fig. 1C).

Notably, the Ile^550^Val mutation occurred as a natural variant during the cloning of NaDC1 from a cDNA library, which was subsequently used to determine the protein’s structures (*12*, *35*). To investigate whether this variant could affect transport activity, we assessed the transport activity by measuring the currents associated with sodium citrate cotransport when NaDC1 was expressed in human embryonic kidney (HEK) 293T cells. The results indicated that the wild-type (WT) NaDC1 is indeed capable of transporting citrate, consistent with previous reports (*36*–*39*). This finding was further confirmed by a dose-dependent transport assay (Fig. 1, D and E). Notably, the Ile^550^Val mutant exhibited comparable transport activity to that of WT NaDC1 (fig. S10, A and B).

### Substrate binding modes of NaS1 and NaDC1

**Fig. 2.**
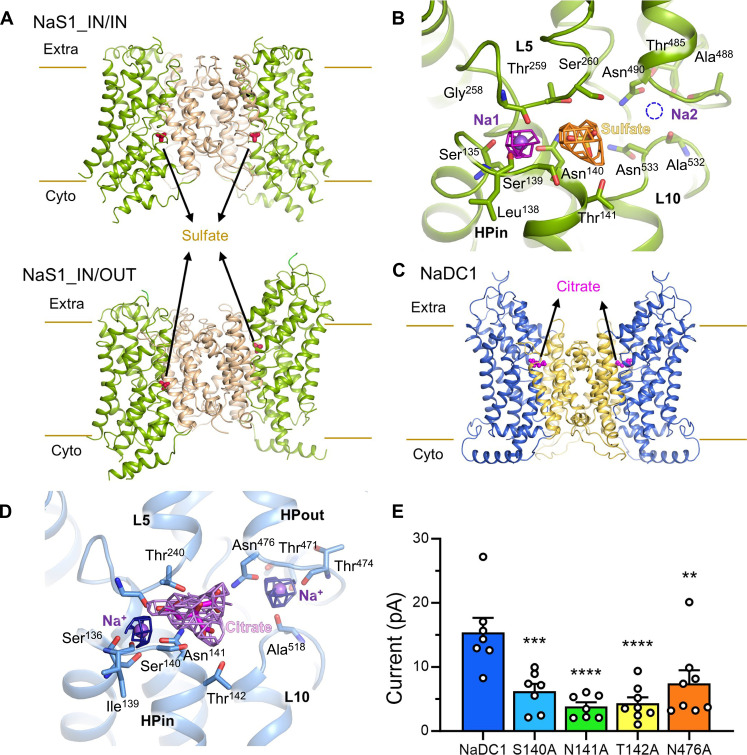
Substrate recognition in NaS1 and NaDC1. (**A**) Sulfate binding in NaS1_IN/IN and NaS1_IN/OUT conformations. The sulfate is coordinated by core domain of NaS1, which remains unchanged in different conformational states. (**B**) Cryo-EM density of sulfate in NaS1_IN/IN. Key residues participating in sulfate and sodium binding are from L5 and HPin of NaS1. The sodium density proposed to be bound around L10 and HPout is not found in cryo-EM map density. (**C**) Citrate binding in NaDC1. Citrate molecules are located in the interface of scaffold and core domain. (**D**) Key residues participating in citrate and sodium binding are from L5, L10, HPin, and HPout of NaDC1. The main chain carbonyl group of Ile^139^ and Thr^474^ is involved in coordination of sodium ions. The cryo-EM map density of citrate and sodium ions is represented in mesh. (**E**) Key residue mutations inhibit transport activity of NaDC1, currents induced by 10 mM citrate, **P < 0.01, ***P < 0.001, and ****P < 0.0001, compared with WT-NaDC1 and calculated by one-way analysis of variance (ANOVA). Data present means ± SEM.

The intricate mechanism governing substrate selectivity between NaSs and NaDCs is elucidated through our high-resolution structural insights (Fig. 2 and fig. S11). Compared with NaS1_apo_IN/IN, there is obvious sulfate density around the classical substrate binding pocket of NaS1_sulfate_IN/IN (Fig. 2, A and B, and fig. S11A). In the structure of NaS1_IN/IN (Fig. 2A, top), sulfate stabilization relies on the side chains of Ser^139^ and Thr^141^ from HPin, as well as Thr^259^ and Ser^260^ of L5 (Fig. 2B). A single sodium ion (named Na1) is distinctly coordinated within the NaS1 structure, involving the carbonyl oxygen atoms of Ser^135^ and Leu^138^, along with the side chain of Asn^140^ from HPin, and the carbonyl oxygen atoms of Gly^258^ on L5 (Fig. 2B). This Na1 site is equivalent to that of NaCT and *Vc*INDY (fig. S11, F to I) (*30*, *32*, *34*), while there is no obvious ion density around equivalent Na2 site NaS1_IN/IN (Fig. 2B). An interesting observation is that the coordination of sulfate in NaS1_IN/OUT conformation, orchestrated by the core domain, remains consistent in both inward- and outward-facing conformations (fig. S11, B to D), thus affirming the unchanging nature of substrate transfer across the elevator transport cycle. Furthermore, the NaS1_IN/OUT conformations imply that each protomer within the dimer can independently execute the substrate transport cycle within SLC13 members. The absence and presence of the two sodium ions in Na1 and Na2 sites also vary in different NaS1 conformations, which, to some extent, reflect different transport steps (fig. S11, A to C).

Shifting focus to the NaDC1_citrate, we discern two sodium ions and one citrate molecule distinctly situated within a single protomer (Fig. 2, C and D, and fig. S11E). A citrate molecule is observed in the substrate pocket similar to NaS1 (Fig. 2C and fig. S11F), which is coordinated by hydrogen bonds with the side chains of Ser^140^ and Thr^142^ from HPin, along with Thr^240^ from L5 (Fig. 2D). Two bulks of ion densities can be determined in the cryo-EM map, locating in the Na1 and Na2 sites of NaDC1. Na1’s coordination involves the carbonyl oxygen atoms of Ser^136^ and Ile^139^, along with the side chain of Asn^141^ from HPin, and the carbonyl oxygen atoms of Gly^239^ on L5. Similarly, Na2’s coordination entails the carbonyl oxygen atoms of Thr^471^ and Thr^474^, alongside the side chain of Asn^476^ from HPout, and the carbonyl oxygen atoms of Ala^518^ on L10 (Fig. 2D). To be noticed, most residues involved in the substrate recognition and sodium coordination are conserved in NaDC1 and NaS1 (fig. S11F). To examine the transport activity, mutations were introduced in NaDC1 by replacing each of these residues with Ala, including the Ser^140^Ala, Asn^141^Ala, Thr^142^Ala, and Asn^476^Ala. All mutants resulting in a marked decrease in citrate-induced currents compared to the WT-NaDC1 with equal level of expression were verified by confocal imaging (Fig. 2E and figs. S10, C and D, and S11K).

To further characterize the role of these residues in NaDC1, we set up the circular dichroism (CD) spectroscopy assay to measure the melting temperature (*T*_m_) in the presence of citrate. The *T*_m_ for WT-NaDC1 is 62.9° ± 0.3°C, while the *T*_m_ values are reduced to 57.1° ± 0.7°C for Ser^140^Ala mutant and 56.4° ± 0.3°C for Thr^142^Ala, respectively. We also found the Asn^141^Ala (61.3° ± 0.5°C) and Asn^476^Ala (60.5° ± 0.7°C) only show a minor reduced *T*_m_ (fig. S12). These results further support the role of Ser^140^ and Thr^142^ in the recognition of citrate.

Notably, a comparative assessment of the substrate binding sites among NaS1, NaDC1, NaCT, and *Vc*INDY reveals notable differences (fig. S11, F to J). A notable variation is observed in Ser^260^ of NaS1, which is corresponding to the Pro^201^ in *Vc*INDY, Gly^288^ in NaCT, and Ala^241^ in NaDC1 of L5, where Ser^260^ in NaS1 could potentially preclude citrate and succinate binding. The Asn^533^ in NaS1, corresponding to Thr^519^ in NaDC1, Thr^508^ in NaCT, and Thr^421^ in *Vc*INDY, might also participate in the substrate selection. In addition to that, the Ile^139^ (NaDC1)/Leu^138^ (NaS1) and Thr^474^ (NaDC1)/Ala^488^ (NaS1) feature main chain carbonyl groups involved in sodium coordination, making mutations inconsequential (Fig. 2, B and D, and fig. S11, F to J). In summary, our findings meticulously unravel the intricate details of substrate recognition for NaS1 and NaDC1, offering vital insights into substrate selectivity mechanisms.

### The allosteric inhibition of NaDC1

**Fig. 3.**
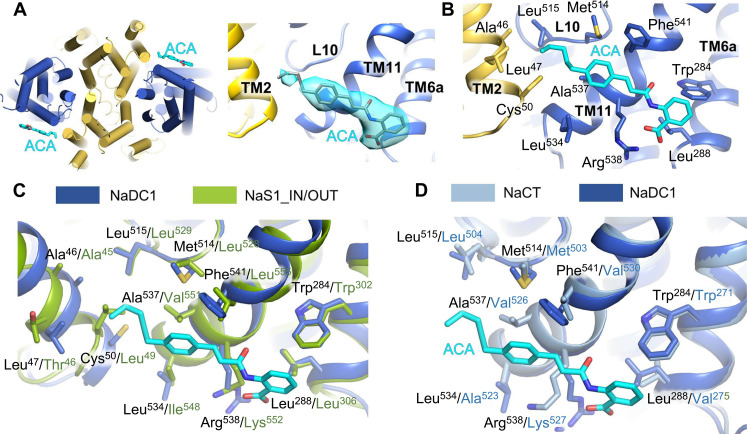
Molecular basis for ACA binding in NaDC1. (**A**) ACA binding pockets in NaDC1. ACA is colored in cyan and located in the peripheral hydrophobic region of NaDC1. The model of ACA is well fitted to the density, which is bound to a hydrophobic pocket mainly formed by TM2 and TM11. Scaffold domain is colored in yellow, and core domain is colored in blue. (**B**) The interface of ACA binding to NaDC1 is mostly contributed by hydrophobic residues from TM2, L10, TM11, and TM6a. which is further enhanced by salt bridge between Arg^538^ and carboxyl group of ACA. The coordination of scaffold domain and core domain is the possible explanation of ACA inhibitory mechanism. (**C**) Comparison of the NaDC1_ACA and NaS1_IN/OUT around the ACA binding pocket. The NaDC1 structure is aligned to the OUT protomer of NaS1_IN/OUT. Most residues in ACA binding remain similar. The major differences lie in Leu^47^ (NaDC1) and Thr^46^ (NaS1), Met^514^ (NaDC1) and Leu^528^ (NaS1), Arg^538^ (NaDC1) and Lys^552^ (NaS1). (**D**) Comparison of the structure of NaDC1 and NaCT around the ACA binding pocket. The NaDC1 structure is aligned to the core domain of NaCT. All key residues in ACA binding remain the same or similar.

Flufenamate, a nonsteroidal anti-inflammatory drug and a kind of anthranilic acids, was identified as human NaDC1 inhibitor with a median inhibitory concentration of ~2 mM (*36*). ACA was found when searching for NaDC1 inhibitors of higher binding affinities, with a median inhibitory concentration lower than 15 μM (*20*). The inhibitory effect of ACA on NaDC1 citrate transport activity was confirmed by our functional assay. A significant decrease in citrate-induced current was detected when ACA was added, and the recovery was observed when ACA was washed (fig. S13A). In search of the molecular basis of ACA inhibition mechanism of NaDC1, the cryo-EM structure of NaDC1 bound with ACA was solved. The ACA molecule was meticulously accommodated within the density (Fig. 3A). The structural comparison of apo, citrate-bound, and ACA-bound forms of NaDC1 revealed a remarkable consistency in their overall architecture (fig. S13B). ACA was demonstrated an affinity for a hydrophobic pocket primarily constituted by hydrophobic residues originating from TM2, L10, TM11, and TM6a (Fig. 3A) and the interaction with NaDC1 being bolstered by a salt bridge formation between Arg^538^ and the carboxyl group of ACA (Fig. 3B and fig. S13C). Notably, the coordination between the scaffold domain and core domain emerges as a plausible rationale for the inhibitory mechanism exerted by ACA.

Previous reports have highlighted ACA’s capacity to hinder the transport activity of NaDC1 and NaCT (*20*). Nonetheless, whether ACA extends its inhibitory effects to NaS1 remained unresolved. A structural alignment of the outward-facing protomer of NaS1_IN/OUT with NaDC1 in the vicinity of the ACA binding site indicated a noteworthy resemblance in most of the residues involved in ACA binding (Fig. 3C and fig. S13D). However, notable disparities were identified, primarily involving Leu^47^ (NaDC1) and Thr^46^ (NaS1), Met^514^ (NaDC1) and Leu^528^ (NaS1), and Arg^538^ (NaDC1) and Lys^552^ (NaS1) (Fig. 3C). Specifically, the hydrophilic nature of Thr^46^ in NaS1 could potentially interfere the hydrophobic pocket formation of TM2, TM11, and L10, hence reducing ACA binding (Fig. 3C).

Furthermore, a comparison of the ACA binding site within the structures of NaDC1 and NaCT unveiled little divergence in the alignment (Fig. 3D). Specifically, the NaDC1 structure was superimposed onto the core domain of NaCT. Most critical residues implicated in ACA binding exhibited consistent or analogous spatial arrangements. Differences were discerned in Arg^538^ (NaDC1) and Lys^527^ (NaCT), as well as Phe^541^ (NaDC1) and Val^530^ (NaCT). The similarity of critical residues in ACA binding could contribute to the ACA sensitivity of NaCT (*20*).

### Putative role of lipid binding in NaS1 and NaDC1

**Fig. 4.**
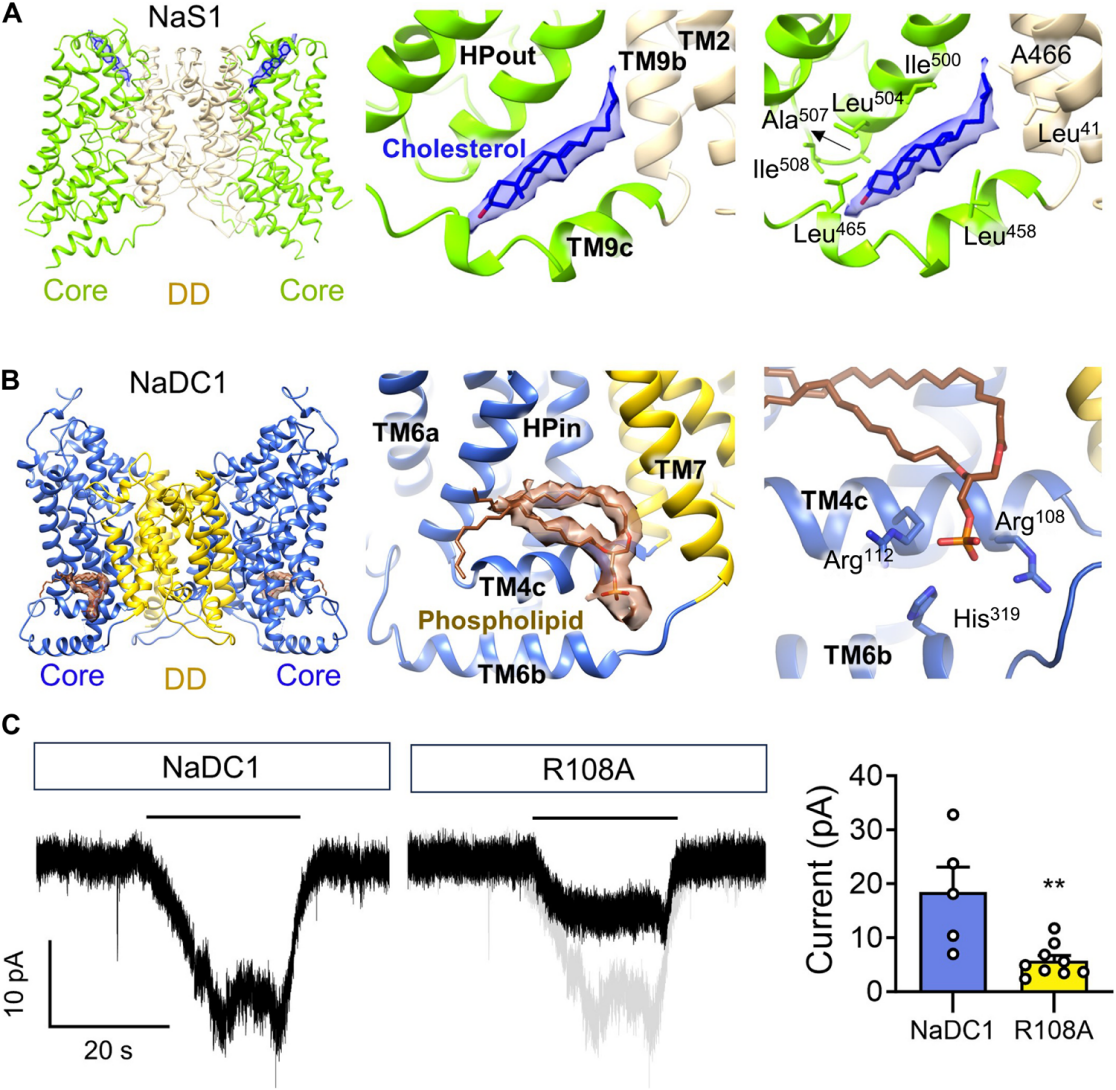
Lipid binding in NaDC1 and NaS1 structures. (**A**) A cholesterol-like molecule steps in the narrow groove between HPout and TM9c of NaS1. Cholesterol was built considering the physiological relevance. Cholesterol is stabilized through hydrophobic interaction with alanines, leucines, and isoleucines from TM9c, HPout, TM9b, and TM2. (**B**) Phospholipid-like molecules are attached to the side of HPin, TM4c, TM6b, and TM7 of NaDC1. The branched tail indicates phospholipids. The polyalkylated tail is bound to the hydrophobic pocket of HPin and TM4c. It is further stabilized throughelectrostatic interaction between polar head of phospholipid and alkaline residues Arg^112^, His^319^, and Arg^108^. (**C**) Arg^108^ mutations hamper transport activity of NaDC1. **P < 0.01, calculated by *t* tests. Data present means ± SEM.

A distinctive feature of our study is the identification of intriguing lipid-binding sites within the structures of NaS1 and NaDC1, unraveling the molecular basis underlying these interactions. In particular, an elongated density emerged within a cleft enclosed by the transmembrane helices HPout, H9c, TM9b, and TM2 of NaS1, where an array of hydrophobic interactions was evident (Fig. 4A and fig. S13E). While this density could conceivably correspond to glyco diosgenin (GDN), given its likely introduction during protein purification, its conformation implies the potential accommodation of a physiologically relevant cholesterol molecule (Fig. 4A).

Furthermore, we unearthed distinct phospholipid-like densities associated with individual protomers on the intracellular side of the membrane in NaDC1 (Fig. 4B and fig. S13F). These densities were enclosed by the HPin, H4c, TM6b, and TM7 regions. The polyalkylated tail of the lipid was firmly lodged within the hydrophobic pocket formed by HPin and TM4c, with additional stabilization facilitated through electrostatic interactions involving alkaline residues Arg^112^, His^319^, and Arg^108^ (Fig. 4B). For illustrative purposes, we modeled a phosphatidic acid at this site; however, the precise phospholipid species necessitates further elucidation. The R108A mutant substantially reduced the citrate-induced current than WT in NaDC1 (Fig. 4C). The coordination of a phospholipid in the NaDC1 structure might serve to underscore the pivotal role of Arg^108^ within H4c, as its mutation has been also demonstrated to abrogate transport functions (*40*).

### Detailed conformational transition in NaS1

**Fig. 5.**
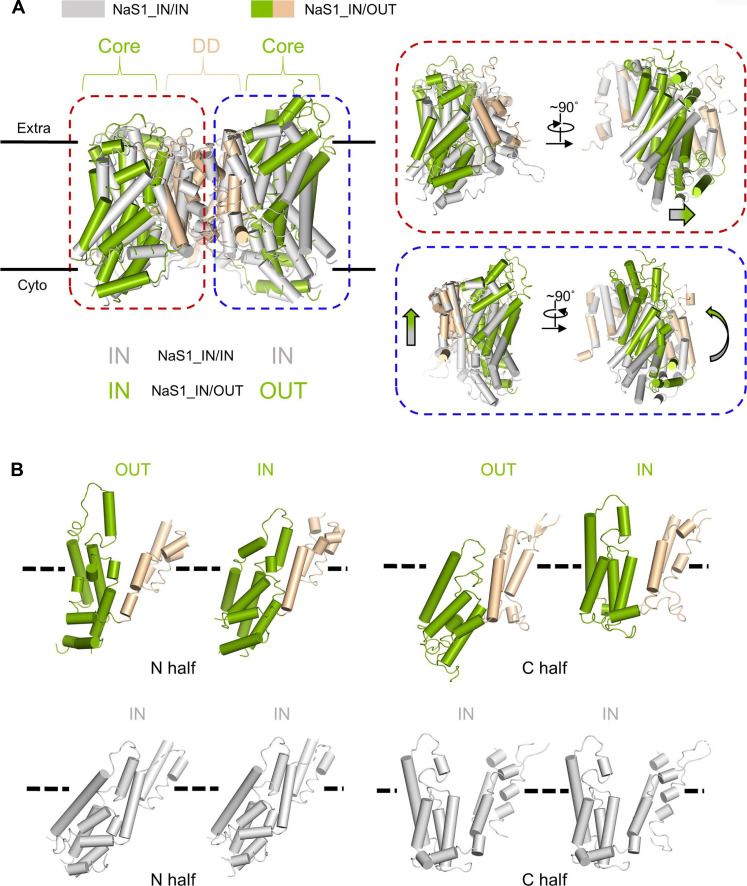
Conformation conversion of NaS1. (**A**) The structures of NaS1-IN/IN and NaS1_IN/OUT are aligned with respect to the dimerization domain. NaS1-IN/IN is colored in gray, and NaS1_IN/OUT is colored green (core domain) and wheat (dimerization domain). Dimerization domain mostly remains unchanged during conformation conversion from inward to outward open. The conformational change of core domains is shown in the right panels. Small movement horizontal to the membrane is detected when comparing the IN subunits of NaS1-IN/IN and NaS1_IN/OUT (encircled by red dashed line). When compared the IN subunits of NaS1-IN/IN and OUT subunits of NaS1_IN/OUT, both horizontal and vertical movements are detected (encircled by blue dashed line). (**B**) The N and C halves of each subunit from NaS1-IN/IN and NaS1_IN/OUT are depictured separately. N and C halves in one subunit exchange their conformations during conformation conversion.

The distinct inward-facing and outward-facing conformations revealed by NaS1 provide important insights into the intricate conformational dynamics underlying the transport cycle (Fig. 5 and fig. S14). In this context, it is remarkable that TM1 to TM3 and TM9 of the dimerization domain maintain its structural integrity throughout the conformational change process, only minor changes of TM4, TM7, and TM8 are detected, emphasizing its role as a stable scaffold. However, the core domain, a pivotal player in the conformational transition, undergoes a notable movement. Upon juxtaposing the IN subunits of NaS1-IN/IN and NaS1_IN/OUT, a subtle yet noteworthy horizontal displacement within the plane of the membrane comes to the fore. This phenomenon, occurring within the core domain, underscores the sensitivity of even minor structural shifts to the transport cycle. Furthermore, a more intricate conformational landscape emerges upon comparing the IN subunits of NaS1-IN/IN with the OUT subunits of NaS1_IN/OUT. This analysis reveals a dual-layered transition involving both horizontal and vertical displacements. This concerted movement spanning two dimensions highlights the concerted nature of the core domain’s reconfiguration (Fig. 5A).

A detailed dissection of the individual subunits, dissected into their N and C halves, further illuminates the conformational transformation during the transport cycle. Notably, the N and C halves within a given subunit undergo a remarkable conformational interchange, a dynamic interplay that mirrors the broader conformational shift of NaS1 during the transport cycle (Fig. 5B).

In summary, our analysis of the NaS1 conformational transition underscores the pivotal role of the dimerization and core domains in orchestrating the transport cycle. The distinct behaviors of the IN and OUT subunits, along with the intricate N and C half exchange, collectively provide a comprehensive pictures of the conformational dynamics underpinning NaS1’s function.

## DISCUSSION

In this study, the elucidation of the structures of NaS1 and NaDC1 marks a notable milestone in our understanding of the transport cycle within the SLC13 member family. Through systematic exploration, we have revealed the complete transport cycle of these transporters, shedding light on the intricate sequence of events governing their function.

**Fig. 6.**
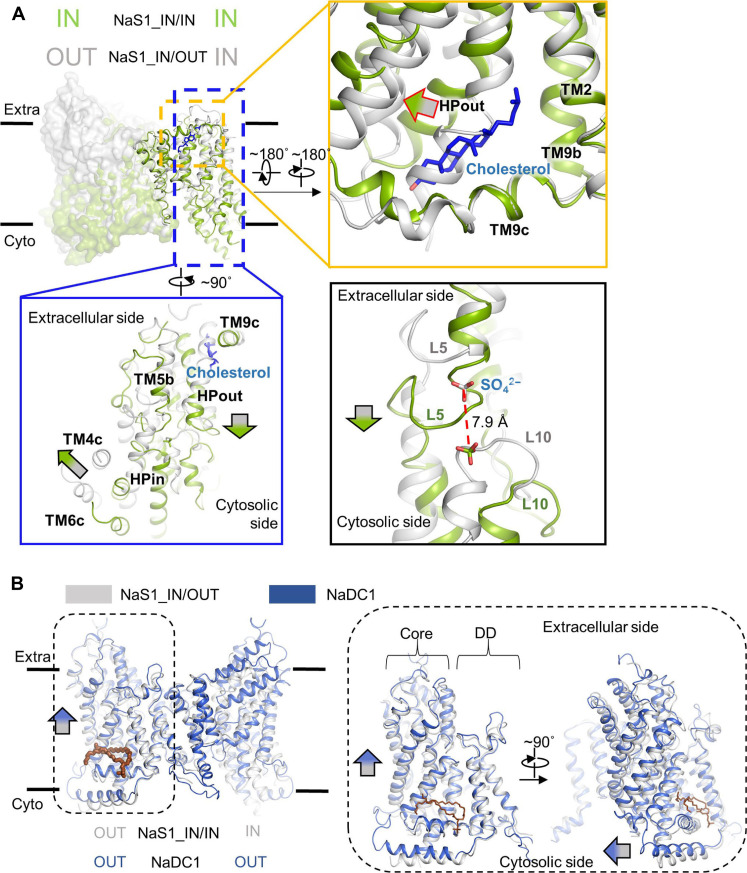
Lipid binding stabilizes the conformation in NaS1 and NaDC1. (**A**) Cholesterol binding stabilizes the inward-open conformation of NaS1. NaS1-IN/IN and NaS1_IN/OUT are aligned to their dimerization domains. The conformation of IN protomer from both structures is compared to illustrate the effect of cholesterol binding. NaS1-IN/IN is colored in green (cholesterol in blue), while NaS1_IN/OUT is colored in gray. The binding of cholesterol in NaS1-IN/IN pushes HPout to move both horizontally and vertically with respect to membrane. The movement is conducted throughout the core domain, inducing large movement of HPin, TM4c, and TM6c in the opposite side. This results in about 7.9 Å movement of substrate toward the cytosolic side. (**B**) Phospholipid binding in NaDC1 slightly pushes the core domain toward extracellular side. NaS1-IN/OUT and NaDC1 were aligned to their dimerization domains (DD). Binding of phospholipid induces the same effect as cholesterol molecule in NaS1 but to the opposite side (movement of core domain to the extracellular side).

A notable finding from the structural analysis is the putative role of cholesterol and phospholipid in regulating the transport function of NaS1 and NaDC1, respectively. When aligned the NaS1-IN/IN and NaS1_IN/OUT structures with their respective dimerization domains (Fig. 6A), we found the potential impact of cholesterol binding. Specifically, the binding pocket in the outward facing conformation of NaS1 could not accommodate the cholesterol molecule. During the transport cycle, the binding of cholesterol within NaS1-IN/IN induces the displacement of HPout in both horizontal and vertical directions with respect to the membrane plane. This movement resonates throughout the core domain, eliciting substantial shifts in HPin, TM4c, and TM6c on the opposite side. This concerted rearrangement culminates in a notable substrate shift of ~7.9 Å toward the cytosolic side (Fig. 6A).

Similarly, aligning NaS1-IN/OUT and NaDC1 structures with their dimerization domains reveals compelling findings (Fig. 6B). In contrast to cholesterol binding to NaS1 C half (HPout and TM9c), phospholipid (modeled as phosphatidic acid) binds to the N half of NaDC1 (HPin and TM4c), which marginally propels the core domain toward the extracellular side. Notably, the binding of the phospholipid elicits an effect analogous to that of cholesterol in NaS1; however, this influence manifests in the opposite direction, causing the core domain to shift toward the extracellular facing state. Distinct lipids induce stabilization of different conformation states of SLC13 family proteins. The profound effects of cholesterol and phospholipid binding on the transport cycle provide important insights into the functional dynamics of these transporters.

It is notable to reveal different lipid coordination in SLC13 family proteins. The different lipids binding in NaS1 and NaDC1 in this study could be the different detergents applied in the protein purification process. While GDN contains a cholesterol-like hydrophobic tail, the enlarged head brought by carbohydrate group cannot fit well into the limited cryo-EM map density. A physiological-related cholesterol is hence built in NaS1 structure. The cholesterol is located in the C half of NaS1. In the similar pocket of NaDC1, N half locates a phospholipid-like molecule. Although cholesteryl hemisuccinate, one of the cholesterol derivatives, was added during protein purification of NaDC1, the phospholipid site is still occupied by endogenous phospholipid-like molecule, indicating a higher affinity toward phospholipid molecules in this site of NaDC1. Lipid binding sites are alike in N and C halves but result in opposite conformational trend of the core domain. The membrane environment is indispensable to transporter activity of SLC13 family members.

Moreover, our structural analyses revealed that substrate binding in inducing conformational changes within proximal regions. In particular, the core domain was observed to execute a rocking motion between the outward-facing and inward-facing conformations throughout the transport cycle. This dynamic behavior underscores the adaptability of these transporters, allowing them to fulfill their critical role in substrate transport. Our investigations have also highlighted the direct modulation of transport activity by lipid molecules such as cholesterol and phospholipids. By virtue of their binding to specific sites, these lipids exert a regulatory influence, contributing to the nuanced control of transporter function.

**Fig. 7.**
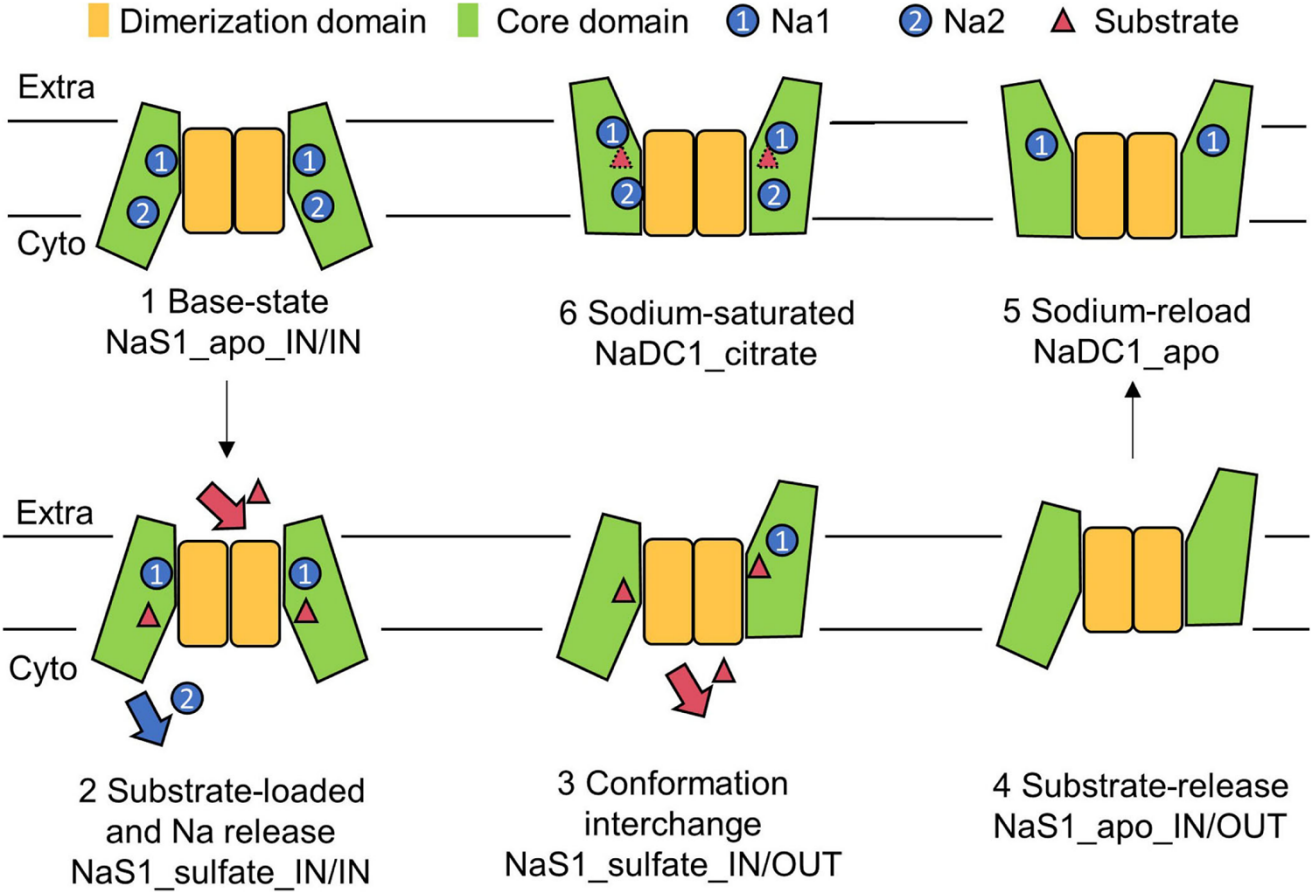
Cartoon scheme of possible conformational conversion in the transport cycle of SLC13 family members. In vertical view of membrane, dimerization domain remains mostly unchanged in the transport cycle. The core domain, in the contrary, moves outward and inward to make the alternative access of the substrates, which supports the elevator model in transmembrane transportation. In the horizontal view of membrane, the movable domains of dimerization domain induce the horizontal movement of core domain. Different subunits in SLC13 family members could act independently in the transport process.

Building upon these findings, we have endeavored to propose a comprehensive working model for the SLC13 family members (Fig. 7 and fig. S14). Initially, the transporter assumes an outward-facing conformation without sodium ions occupying the binding sites (base state). Subsequently, sodium ions from the extracellular fluid occupy the sodium binding sites in the core domain, followed by the substrate binding to the pocket (sodium load and substrate load). The conformational change of the core domain then shifts the substrate’s position to access the intracellular space, resulting in the release of both sodium ions and the substrate (substrate release). Last, the inward-facing conformation may independently undergo a conformational change back to the outward-facing state, thereby could restart the transport cycle (Fig. 7). Each of these states can be represented by one of the structures indicated in the figure. This orchestrated rearrangement effectively transitions the substrate binding site from an outward-facing to an inward-facing state, facilitating substrate release.

Furthermore, our investigation into the allosteric inhibition mechanism has provided valuable insights into the interaction between these transporters and ACA. The constraint induced by ACA between dimerization domain and core domain hampers the relative movement in the transport cycle. The hydrophobic binding site of ACA provides clues to develop allosteric modulatory predrug chemicals for SLC13-related metabolic diseases, including cancer, diabetes, and even neurological disorder (*41*). By discerning structural discrepancies and distinct residue features, we have unveiled the intricate details underlying ACA’s inhibitory effects on NaS1 and NaDC1.

In summary, the high-resolution structures of NaS1 and NaDC1 presented in this study mark a pivotal advancement in our comprehension of the intricate mechanisms governing human SLC13 members. These insights not only enhance our fundamental understanding of these transporters but also offer crucial signposts for the design of therapeutic strategies targeting related diseases. This study represents a stepping stone toward unraveling the intricacies of transporter function and the development of targeted interventions.

## MATERIALS AND METHODS

### Protein expression and purification

The cDNAs of full-length human *SLC13A1* (NP_071889.2, UniProtKB ID: Q9BZW2) and *SLC13A2* (NP_003975.1, UniProtKB ID: Q13183) were cloned into the pCAG vector (Invitrogen) with an N-terminal FLAG tag, respectively. A nature variant I550V was detected in SLC13A2 sequence (*12*). The plasmids used to transfect cells were prepared by the GoldHi EndoFree Plasmid Maxi Kit (CWBIO). The plasmid quality was assessed using the Trans2K Plus DNA Marker (TransGen Biotech).

The recombinant protein was overexpressed using the HEK293F mammalian cells at 37°C under 5% CO_2_ in a Multitron-Pro shaker (Infors; 130 rpm). When the cell density reached 2.0 × 10^6^ cells/ml, the plasmid was transiently transfected into the cells. To transfect one liter of cell culture, about 1.5 mg of the plasmid was premixed with 3 mg of polyethylenimines (Polysciences) in 50 ml of fresh medium for 15 min before adding to cell culture. Cells were collected by centrifugation at 4000*g* for 15 min after 48 hours of transfection.

The cells were collected in buffer containing 25 mM tris (pH 8.0), 150 mM NaCl, and three protease inhibitors, aprotinin (1.3 μg/ml; AMRESCO), pepstatin (0.7 μg/ml; AMRESCO), and leupeptin (5 μg/ml; AMRESCO). For purification of NaS1, the membrane fraction was solubilized at 4°C for 2 hours with 1% (w/v) of GDN (Anatrace), and the cell debris was removed by centrifugation at 18,700*g* for 45 min. The supernatant was loaded to anti-FLAG M2 affinity resin (Sigma-Aldrich). After rinsing with the Wash1 buffer containing 25 mM tris (pH 8.0), 150 mM NaCl, and 0.02% (w/v) of GDN, the protein was eluted with wash buffer plus FLAG peptide (0.2 mg/ml). The eluent was subjected to size exclusion chromatography (Superose 6 Increase 10/300 GL, GE Healthcare) in buffer containing 25 mM tris (pH 8.0), 150 mM NaCl, and 0.01% (w/v) of GDN. For purification of NaDC1, cell was lysed by crude extraction in 1% (w/v) of *n*-dodecyl-β-d-maltoside (Anatrace) and 0.2% (w/v) of cholesteryl hemisuccinate (Anatrace). The supernatant was loaded to anti-FLAG M2 affinity resin (Sigma-Aldrich) after centrifugation at 16,500*g* for 40 min. The resin was washed by Wash2 buffer consisting of 20 mM Hepes (pH 7.4), 150 mM NaCl, and 0.02% (w/v) of GDN. The protein was eluted by FLAG peptide (0.2 mg/ml) and further purified by size exclusion chromatography (Superose 6 Increase 10/300 GL, GE Healthcare) with Wash2 buffer. The respective peak fractions of NaS1 and NaDC1 were verified by SDS–polyacrylamide gel electrophoresis and concentrated for EM analysis.

To obtain the structure of NaDC1 with citrate, the peak fractions of NaDC1 were incubate with 10 mM sodium citrate in 4°C overnight. Then, it is concentrated to around 4 mg/ml for cryo-EM sample preparation. The 10 mM sodium citrate was substituted to 400 μM ACA for obtaining the structure of NaDC1 with ACA.

### Cryo-EM data collection and processing

All protein samples were concentrated to ~4 to 10 mg/ml for Cryo-EM sample preparation. Proteins were applied to glow-discharged Holey Carbon grids (Quantifoil Au 300 mesh R1.2/1.3), which was blotted for 4 to 8 s and then fast-frozen to liquid ethanol through operation on Vitrobot Mark IV (Thermo Fisher Scientific). Data collection was carried out in a 300-kV Titan Krios transmission electron microscope, equipped with Gatan K3 direct electro detector and Gatan GIF Quantum energy filter. AutoEMation is applied for automatic data collection (*42*). The flowchart of data processing of NaS1 and NaDC1 is shown in figs. S2 to S8.

For NaDC1 structures, RELION 3 (*43*) was applied in data processing. The detailed information for data processing can be found in fig. S2. Briefly, particles selected by two-dimensional (2D) classification were first subjected to global angular search 3D classification using RELION 3 with one class. Then, the particles are further classified with global angular search 3D classification with four classes. Good particles were combined and subject to 3D autorefinement, local defocus correction, and postprocessing resulting in a final resolution at 2.72 Å (NaDC1_citrate, 1,369,087 particles), 2.53 Å (NaDC1_apo, 461,481 particles), and 3.28 Å (NaDC1_ACA, 444,389 particles).

For NaS1 structures, CryoSPARC (*44*) was applied in data processing (figs. S4 and S6). Briefly, particles selected by seed facilitated 2D classification were further classified by heterogenous refinement. Two different conformations of NaS1 were found and further refined by nonuniform refinement, respectively. One round of heterogeneous refinement was applied to further extract good particles for NaS1_apo_ state dataset. After nonuniform refine, NaS1_apo__IN/IN reaches 2.93 Å (157,920 particles), NaS1_apo__IN/OUT reaches 2.99 Å (169,571 particles), NaS1_sulfate__IN/IN reaches 3.1 Å (161,063 particles), and NaS1_sulfate__IN/OUT reaches 3.2 Å (151,181 particles).

### Model building and structure refinement

Model building of NaDC1 was performed by Phenix (*45*) and Coot (*46**,* *47*) based on the starting template of *Vibrio cholerae* INDY structure [Protein Data Bank (PDB) ID: 4F35]. Chainsaw in CCP4 (*48*) package was used to substitute the sequence for NaDC1. Each residue was manually checked with the chemical properties considered during model building. Several segments of the sequence were not modeled because of the invisibility of the corresponding density in the map. The model building of NaS1 structures were similar but with starting template of NaDC1. Structure refinement was performed with Phenix with secondary structure and geometry restraints to prevent structure overfitting. To monitor the overfitting of the model, the model was refined against one of the two independent half maps from the gold-standard 3D refinement approach. Then, the refined model was tested against the other map. Statistics associated with data collection, 3D reconstruction, and model building can be found in tables S1 and S2.

### Electrophysiology

HEK293T cells were sustained by Dulbecco’s modified Eagle’s medium (high-glucose, Gibco) containing 10% fetal bovine serum (Gibco) and streptomycin/penicillin (Gibco) at 37°C in 5% CO_2_. Three micrograms of plasmid of SLC13A2 (tagged with green fluorescent protein and Flag) or mutants were transfected by Lipofectamine 2000 (Thermo Fisher Scientific) at a density of ~50% of cells. Forty-eight hours after transfection, the cells were reseeded onto poly-d-lysine–coated coverslips 4 hours before the patch-clamp recordings.

For the whole-cell recording, patch pipettes (3 to 7 megohms) that were fabricated from 1.0-mm capillary glass using a P-97 puller were used to record currents with an EPC-10 USB amplifier (HEKA). The bath solution contains 140 mM NaCl, 5 mM KCl, 2 mM MgCl_2_, 2 mM CaCl_2_, 10 mM glucose, and 10 mM Hepes, adjusted to pH 7.4 with NaOH. The pipette solution contains 126 mM K-gluconate, 10 mM NaCl, 1 mM MgCl_2_, 10 mM Hepes, and 10 mM EGTA, adjusted to pH 7.4 with KOH. The recording chamber (1500 μl) was continuously superfused at 2 ml/min.

### Confocal imaging

The transfected cells that expressing green fluorescent protein–tagged NaDC1 or mutants were fixed with 4% paraformaldehyde for 30 min at room temperature and then washed three times with phosphate-buffered saline. The coverslips were then washed with phosphate-buffered saline and mounted in FluroShield with 4′,6-diamidino-2-phenylindole (Sigma-Aldrich) and observed under a confocal laser scanning microscope LSM 980 (Zeiss, Germany). Each group has three coverslips, and two pictures were taken from each cover glass. ImageJ was used for the quantification.

### CD spectroscopy analysis

CD measurements were performed with the Applied Photophysics Chirascan CD spectrometer. The loss of secondary structure of protein was observed by recording the CD signal at 222 nm as a function of temperature. During the experiment, the temperature of the sample changed at a rate of ~1°C/min between 20° and 98°C. The temperature of the sample was controlled during the measurements by a sensor built into the cuvette holder. The sensor was connected to a circulating bath that adjusted the temperature of the sample. The temperature scale in the final plots represents the temperature actually measured by the sensor in the sample. The CD signal was converted into a ratio of protein folding. Fitting the protein folding ratio versus temperature with a sigmoidal function showed the apparent melting temperature at the midpoint of the transition (*T*_m_).
