# Supplementary material for: Cryo-EM structures of the human NaS1 and NaDC1 transporters revealed the elevator transport and allosteric regulation mechanism
Source: Sci Adv. 2024 Mar 29;10(13):eadl3685. doi: 10.1126/sciadv.adl3685 (PMC10980263; doi:10.1126/sciadv.adl3685)
Supplement: Supplementary file 1 — Figs. S1 to S14 Tables S1 and S2 [file sciadv.adl3685_sm.pdf]

Science Advances
AAAS

Supplementary Materials for

Cryo-EM structures of the human NaS1 and NaDC1 transporters revealed the elevator transport and allosteric regulation mechanism

Ximin Chi *et al.*

Corresponding author: Ximin Chi, ciemen2014@outlook.com; Zilong Wang, wangzl6@sustech.edu.cn; Renhong Yan, yanrh@sustech.edu.cn

Sci. Adv. **10**, eadl3685 (2024)
DOI: 10.1126/sciadv.adl3685

This PDF file includes:

Figs. S1 to S14
Tables S1 and S2

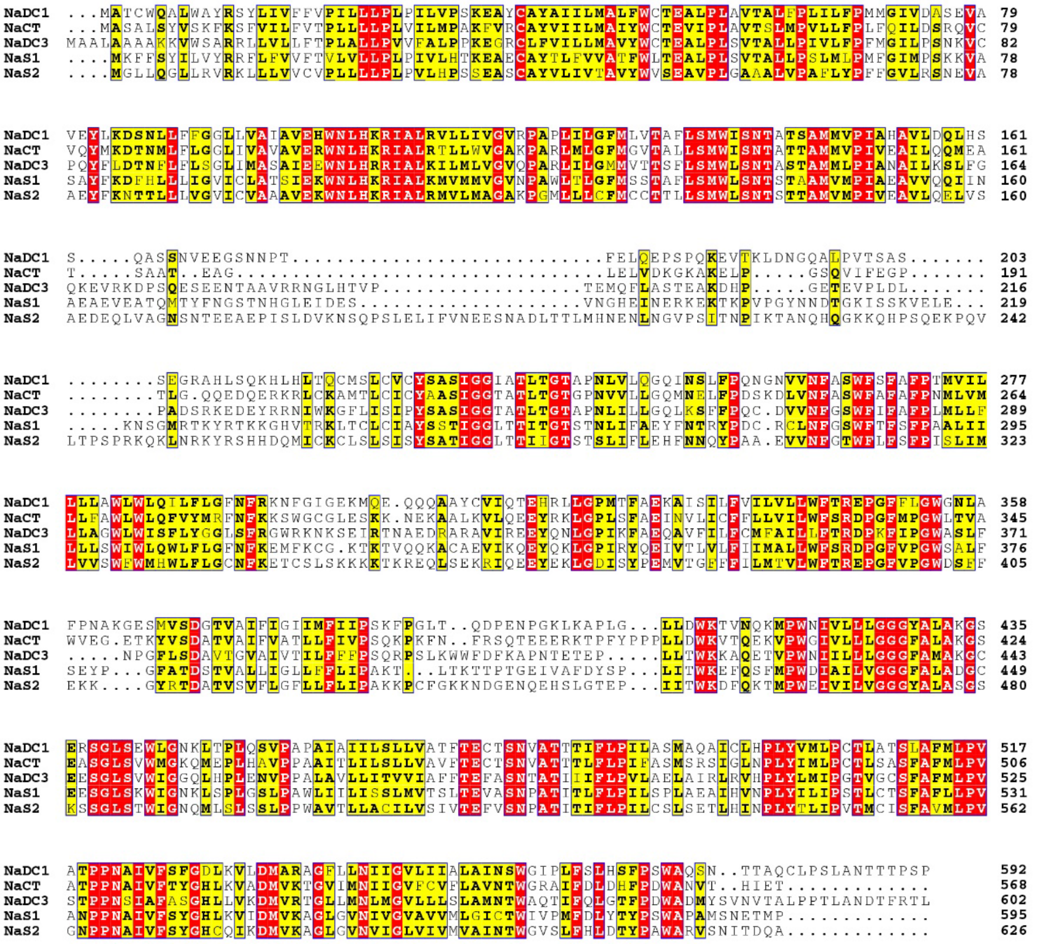

Fig.S1. Sequence alignment of the homologs of SLC13 family from *homo sapiens*. The five sequences are aligned using ClustalX. Identical and conserved residues are shaded red and yellow, respectively. The Uniprot IDs for the proteins aligned are: NaDC1: Q13183; NaCT: Q86YT5; NaDC3: Q8WWT9; NaS1: Q9BZW2; NaS2: Q9UKG4.

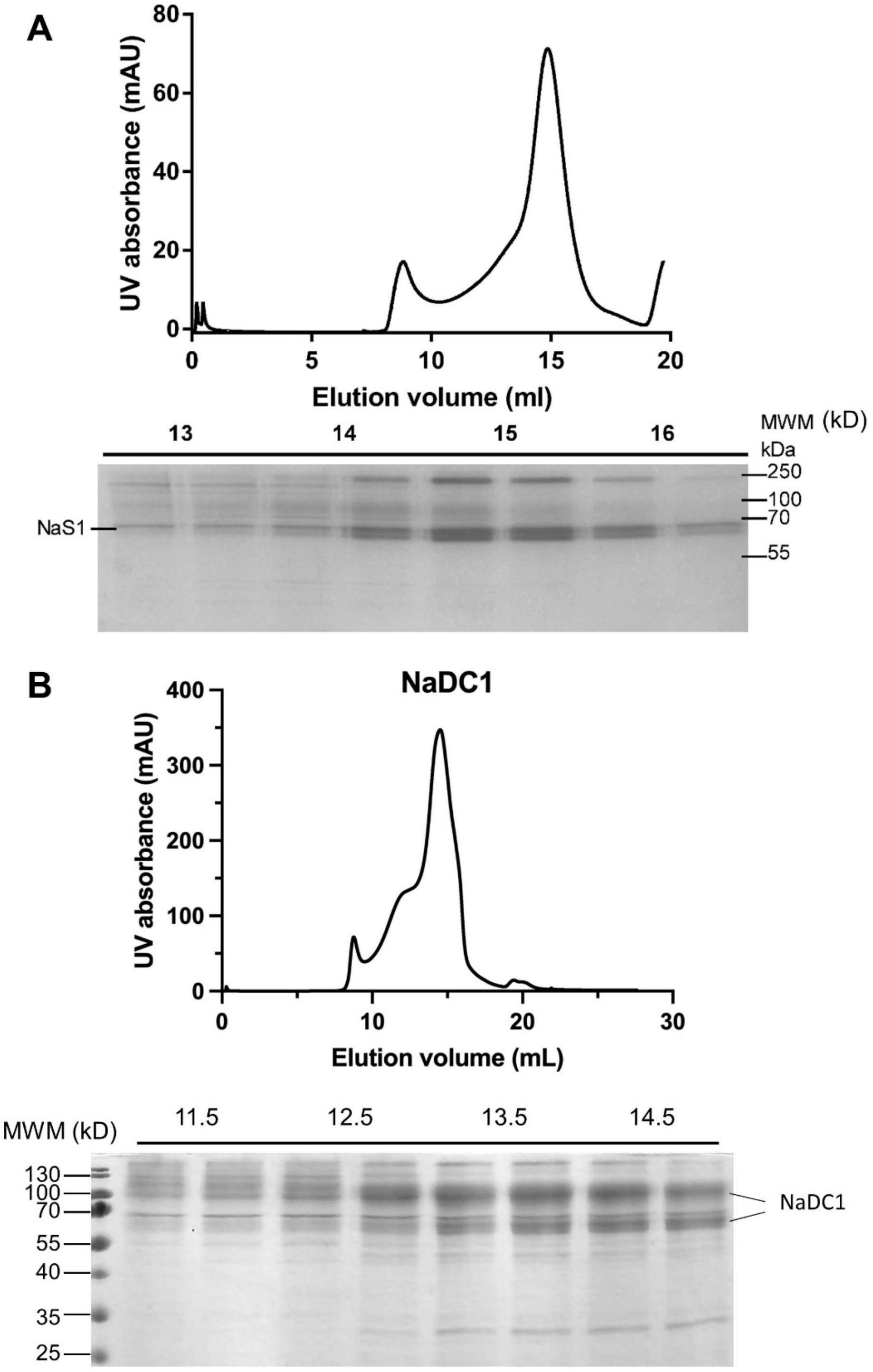

Fig. S2. Biochemical characterization of NaS1 and NaDC1. (**A**) Representative SEC purification of NaS1. (**B**) Representative SEC purification of NaDC1. SDS–PAGE was visualized by Coomassie blue staining.

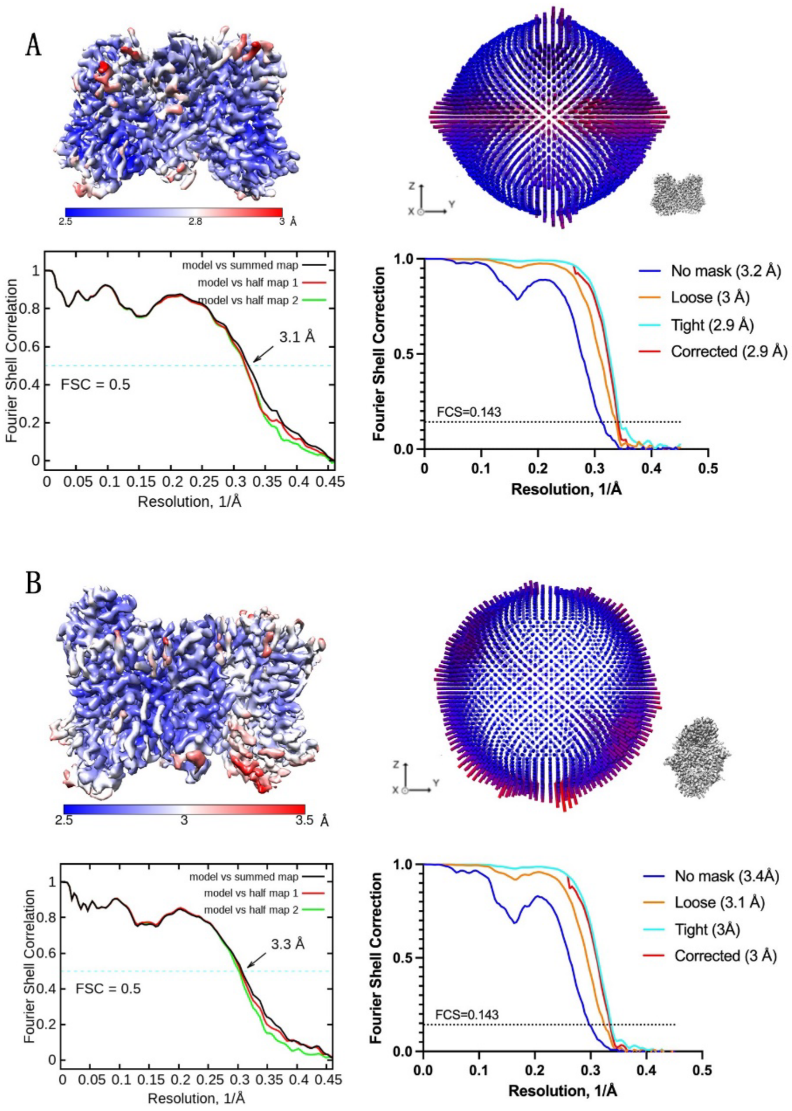

Fig. S3. Cryo-EM analysis of the NaS1 apo state. (**A**) Local resolution map and Euler angle distribution for the 3D reconstruction of NaS1 apo state ($NaS1_{apo}$-IN/IN) are shown in the upper two panels. The left lower panel is the FSC curve of the refined model of NaS1 versus the cryo-EM map that it is refined against (black); of the model refined against the first half map versus the same map (red); and of the model refined against the first half map versus the second half map (green). The small difference between the red and green curves indicates that the refinement of the atomic coordinates did not suffer from overfitting. The right lower panel is the Gold standard FSC curve for the 3D refinement of the $NaS1_{apo}$-IN/IN state. (**B**) same as a but for NaS1 apo state ($NaS1_{apo}$-IN/OUT).

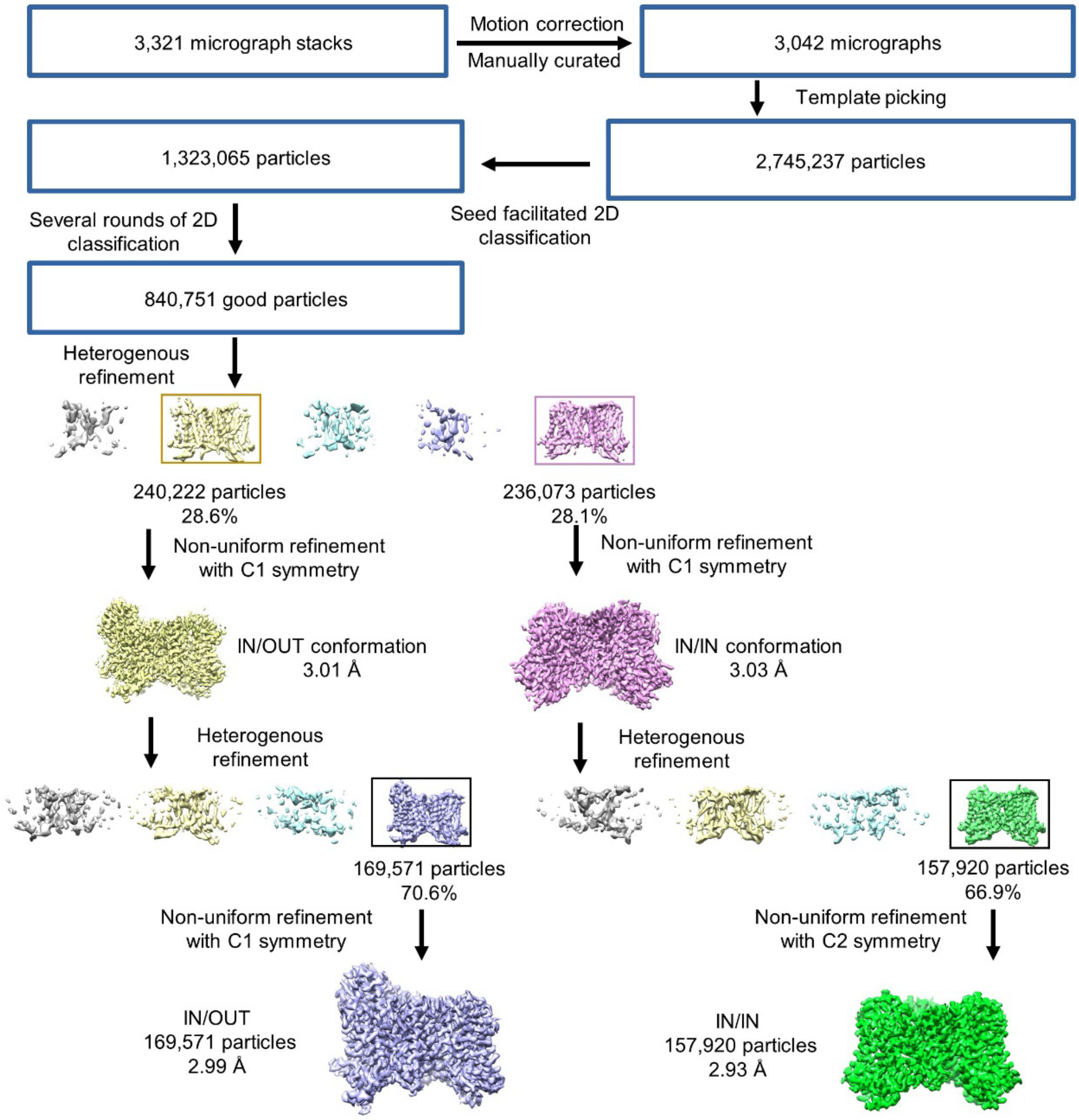

Fig. S4. Flowchart for cryo-EM data processing of NaS1 apo state structures. Please refer to the Materials and Methods, Fig. S5 and Table S2 for details.

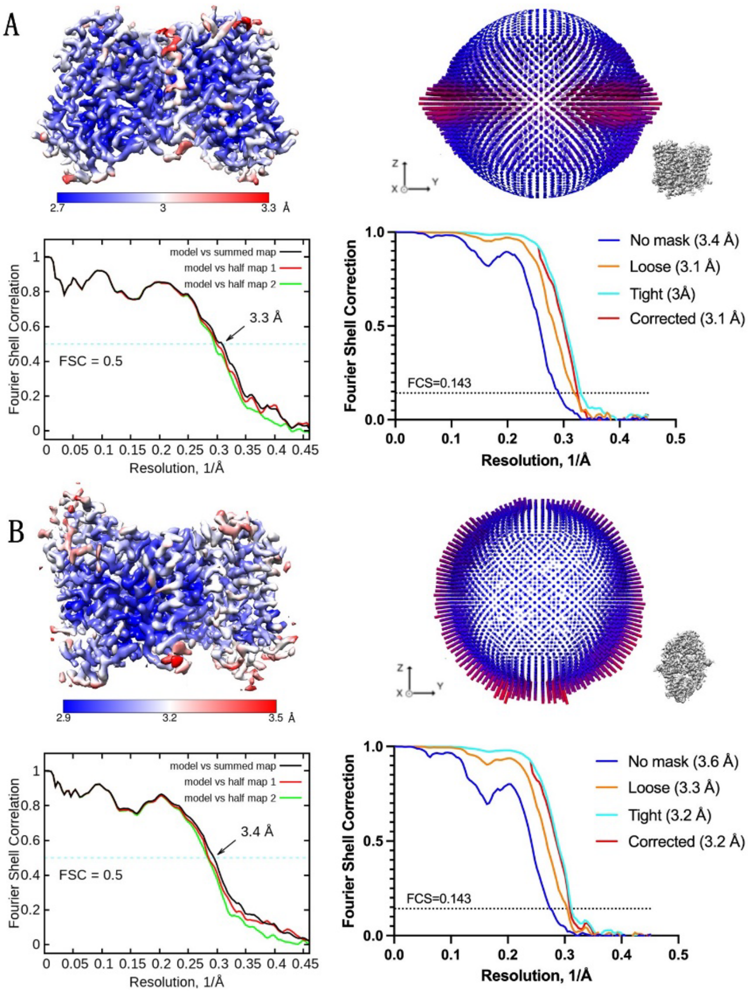

Fig. S5. Cryo-EM analysis of the NaS1 in complex with sulfate. (**A**) Local resolution map and Euler angle distribution for the 3D reconstruction of NaS1 binding to sulfate ($NaS1_{sulfate}$-IN/IN) are shown in the upper two panels. The left lower panel is the FSC curve of the refined model of NaS1 versus the cryo-EM map that it is refined against (black); of the model refined against the first half map versus the same map (red); and of the model refined against the first half map versus the second half map (green). The small difference between the red and green curves indicates that the refinement of the atomic coordinates did not suffer from overfitting. The right lower panel is the Gold standard FSC curve for the 3D refinement of the $NaS1_{sulfate}$-IN/IN state. (**B**) same as a but for NaS1 binding to sulfate ($NaS1_{sulfate}$-IN/OUT).

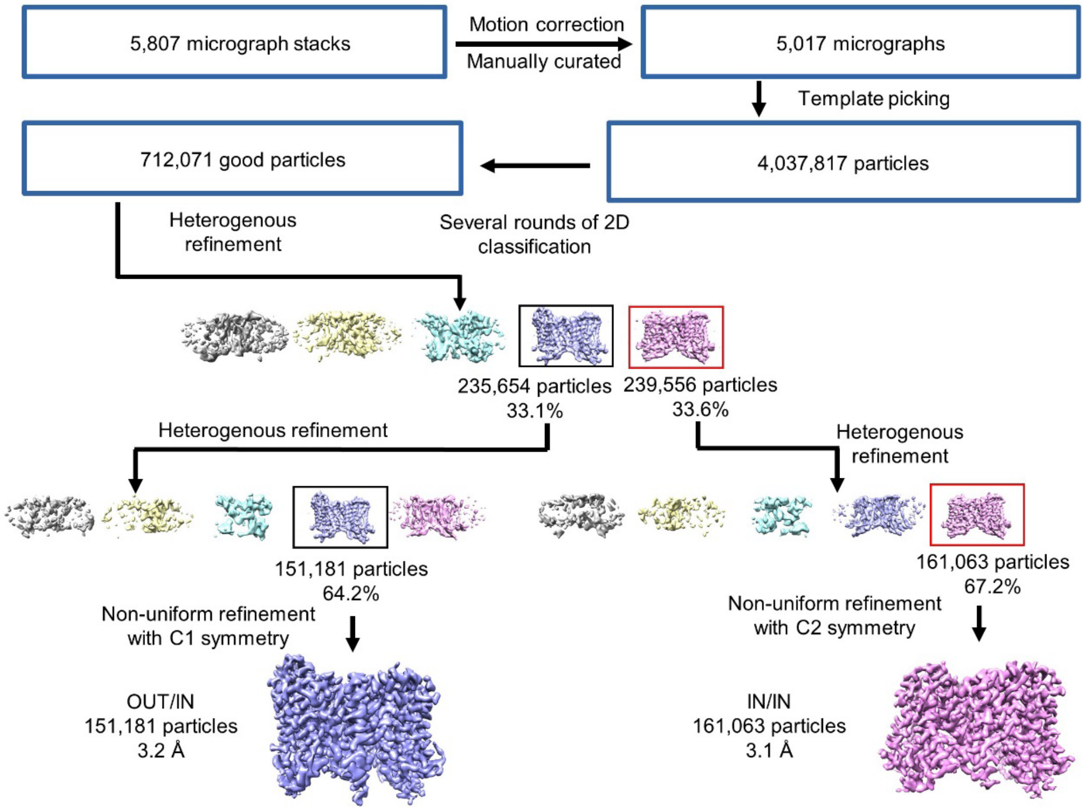

Fig. S6. Flowchart for cryo-EM data processing of NaS1 complex with sulfate structures. Please refer to the Materials and Methods, Fig. S7 and Table S2 for details.

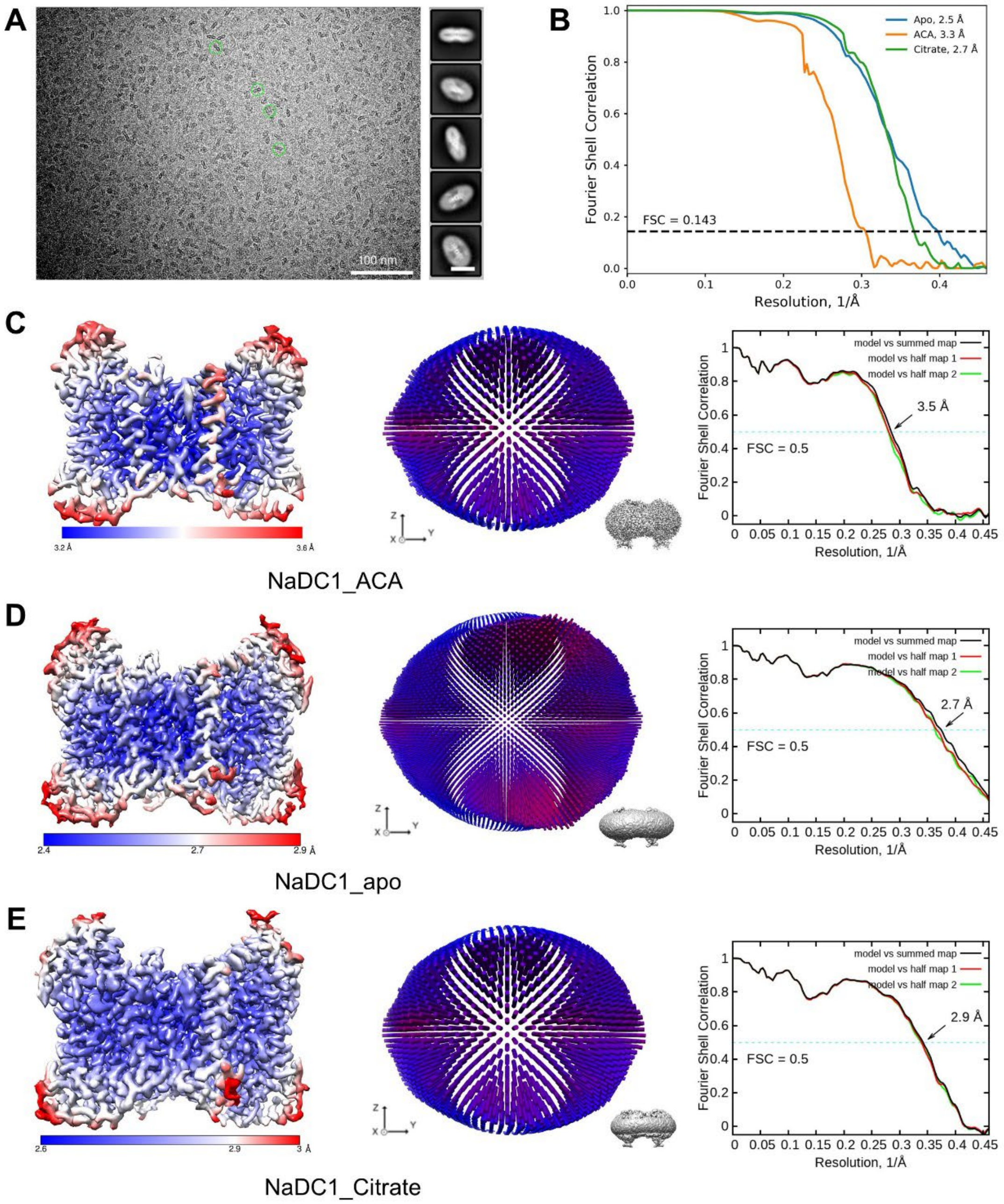

Fig. S7. Cryo-EM analysis of the NaDC1. (**A**) Representative cryo-EM micrograph as well as 2D class averages results. The scale bar in 2D class averages is 10 nm. (**B**) Gold standard FSC curve for the 3D refinement of the NaDC1 apo state (blue), NaDC1 in complex with ACA (orange), and NaDC1 in complex with citrate (green), respectively. (**C**) Local resolution map and Euler angle distribution for the 3D reconstruction of NaDC1 in complex with ACA (NaDC1_ACA) are shown in the left and middle. FSC curve of the refined model of NaDC1_ACA versus the cryo-EM map that it is refined against (black); of the model refined against the first half map versus

the same map (red); and of the model refined against the first half map versus the second half map (green). The small difference between the red and green curves indicates that the refinement of the atomic coordinates did not suffer from overfitting. (**D**) and (**E**) same as c, but for NaDC1 apo state (NaDC1_apo) and NaDC1 with citrate (NaDC1_citrate).

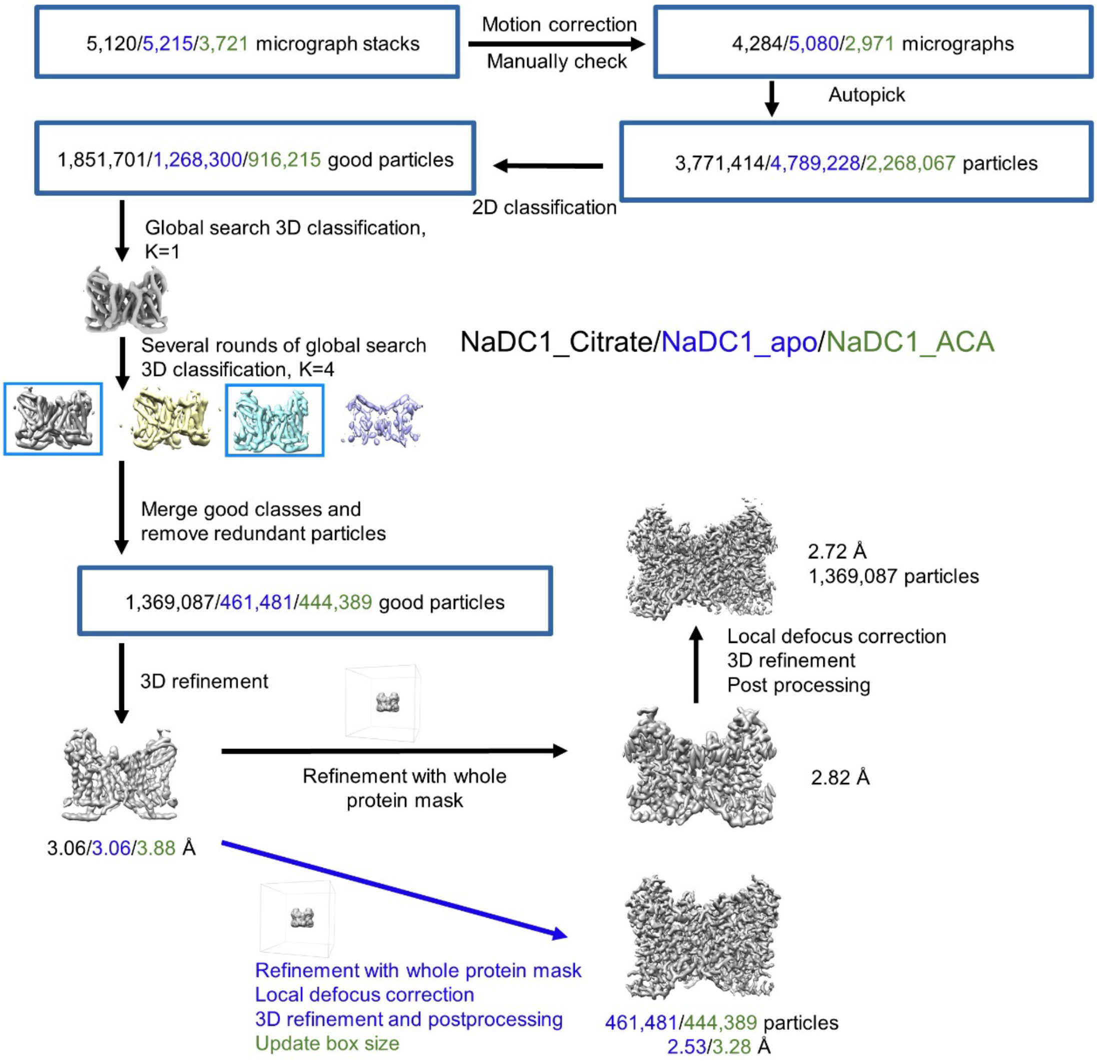

Fig. S8. Flowchart for cryo-EM data processing of NaDC1 structures. Please refer to the Materials and Methods, Fig. S3 and Table S1 for details.

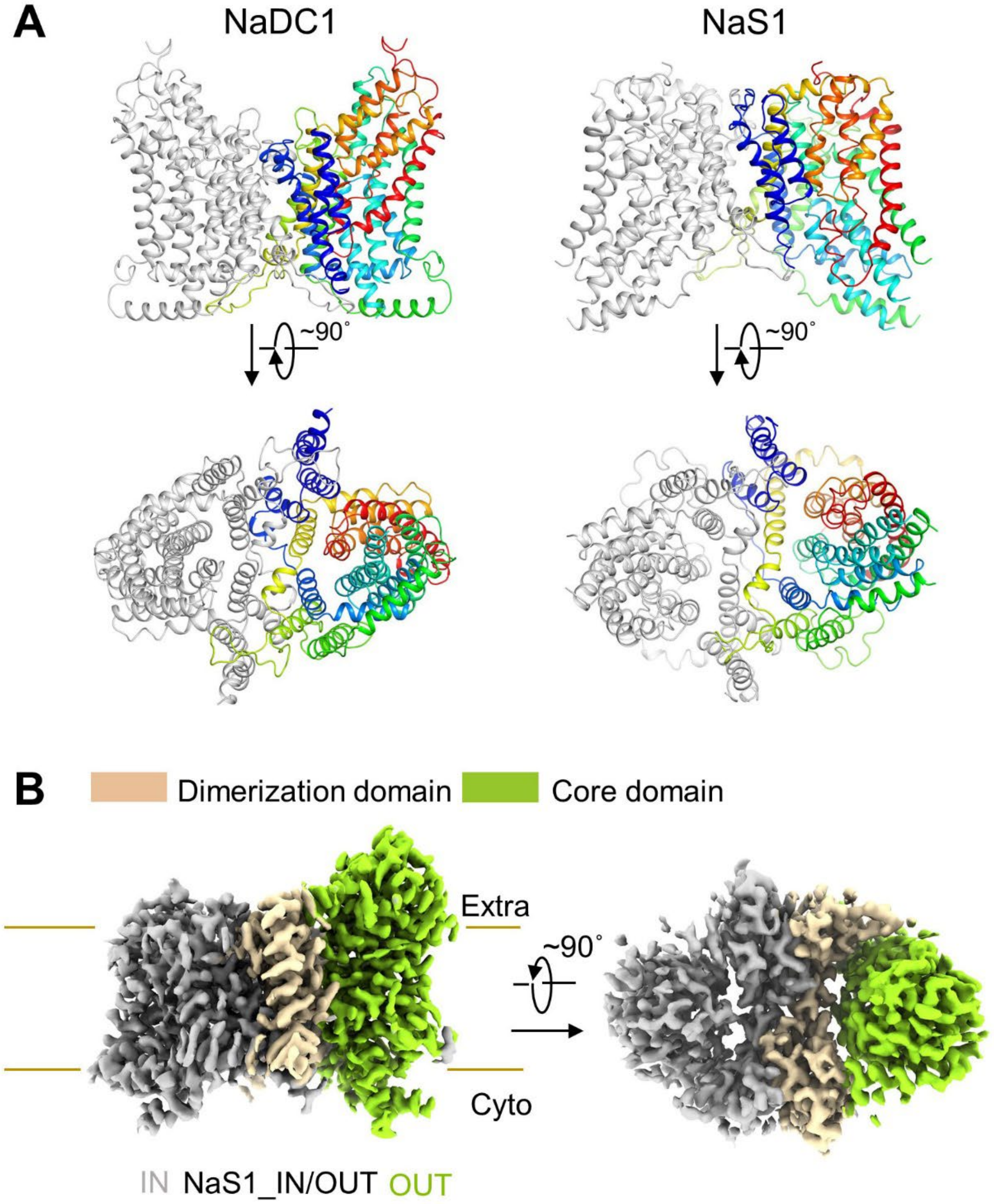

Fig. S9. Cartoon of overall structures of NaS1 and NaDC1. (**A**) Cartoon of overall structures of NaS1 and NaDC1. Only one subunit in each protein is colored in rainbow. (**B**) cryo-EM map of NaS1_IN/OUT. The OUT subunit is colored according to domains.

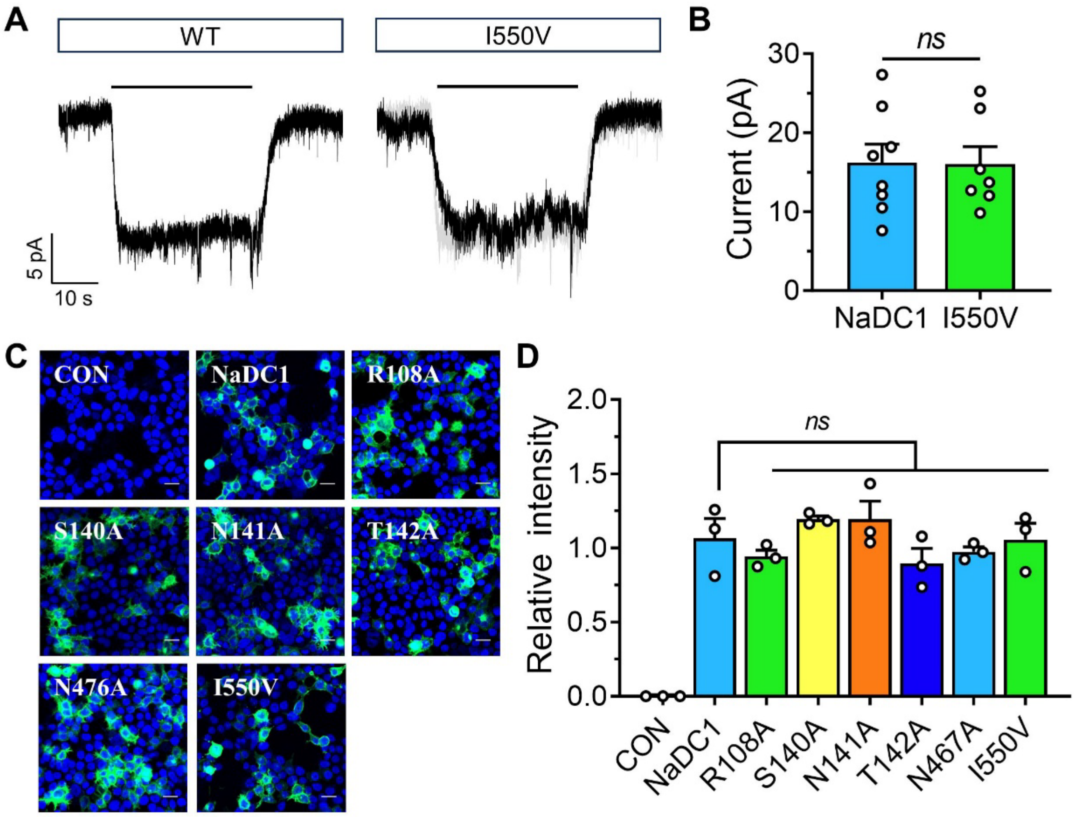

Fig. S10. Confocal images of HEK-293T cells expressing NaDC1 or mutants. (A) Representative traces of currents of WT-NaDC1 and I550V by 10 mM citrate. **(B)** Quantification of currents of WT-NaDC1 and I550V**.** ns, no significance, calculated by t-test. **(C)** Representative images of GFP signaling of NaDC1 and mutants transfected HEK-293T cells. Scale bar = 20 μm. **(D)** Quantification of fluorescence intensity. Note the GFP signal is absent in blank HEK293T cells. ns, no significance, calculated by One-way ANOVA. All data present Mean ± SEM.

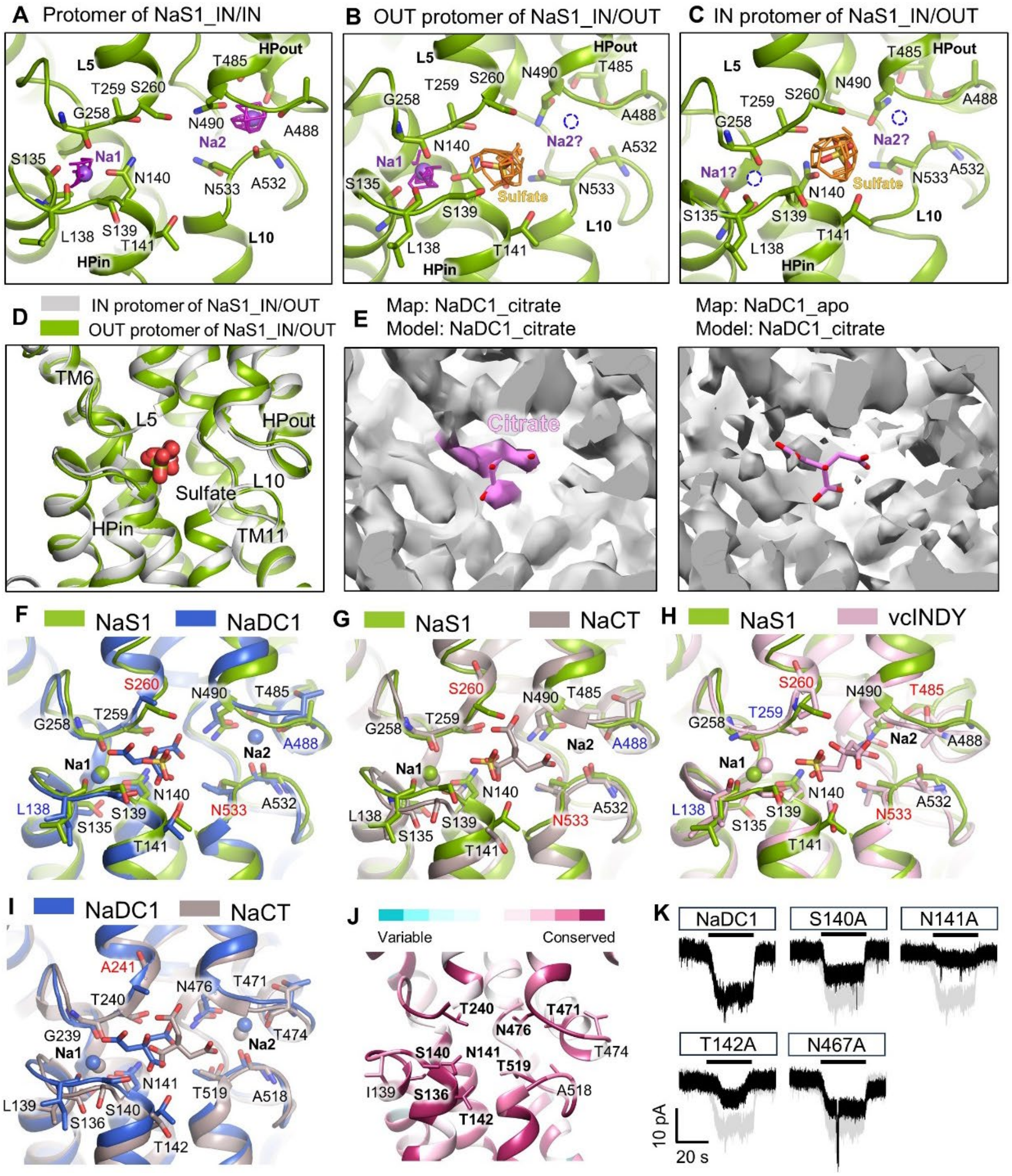

Fig. S11. Substrate binding pockets are highly conserved in SLC13 proteins. (**A**) Sulfate binding pocket in NaS1_apo_IN/IN. The sodium ions are bound to Na1 and Na2 sites, of which the cryo-EM density map are shown in mesh. (**B**) Sulfate binding in outward facing protomer of NaS1_sulfate_IN/OUT. Sodium density near L10 is not obvious in cryo-EM map. (**C**) Sulfate binding in inward facing protomer of NaS1_sulfate_IN/OUT. Both sodium densities are not obvious in cryo-EM map. (**D**) Sulfate binding of different subunits are alike in NaS1_IN/OUT. (**E**) Although the density of citrate is not continuous in cryo-EM map probably due to conformation flexibility, there is no obvious density around citrate binding pocket in NaDC1_apo

structure. (**F**) Comparison of the substrate binding pockets of NaS1 and NaDC1. Most residues involved in substrate coordination are conserved. The major differences lie in S260(NaS1)/A240(NaDC1) and N533(NaS1)/T519(NaDC1). As for I139(NaDC1)/L138(NaS1), T474(NaDC1)/A488(NaS1), main chain carbonyl groups are involved in sodium coordination, thus the mutations are indifferent. Only residues of NaS1 are labled, as also in (**G**) and (**H**). (**G**) Comparison of the substrate binding pockets of NaS1 and NaCT. Major differences lie in S260 (NaS1)/G288(NaCT) and N533(NaS1)/T508(NaCT). (**H**) Comparison of the substrate binding pockets of NaS1 and vcINDY. Major differences lie in S260 (NaS1)/P201(vcINDY), N533(NaS1)/T421(vcINDY), and T485(NaS1)/L372(vcINDY). T259 change to similar residue S200 in vcINDY. (**I**) Comparison of the substrate binding pockets of NaDC1 and NaCT. Only one difference appears in A241(NaDC1)/G288(NaCT). All other residues shown remain unchanged. (**J**) Residues conservation scheme depictured by Consurf. Most residues around the substrate binding sites are well-conserved. (**K**) Representative traces of currents of WT-NaDC1 or mutants induced by 10 mM citrate.

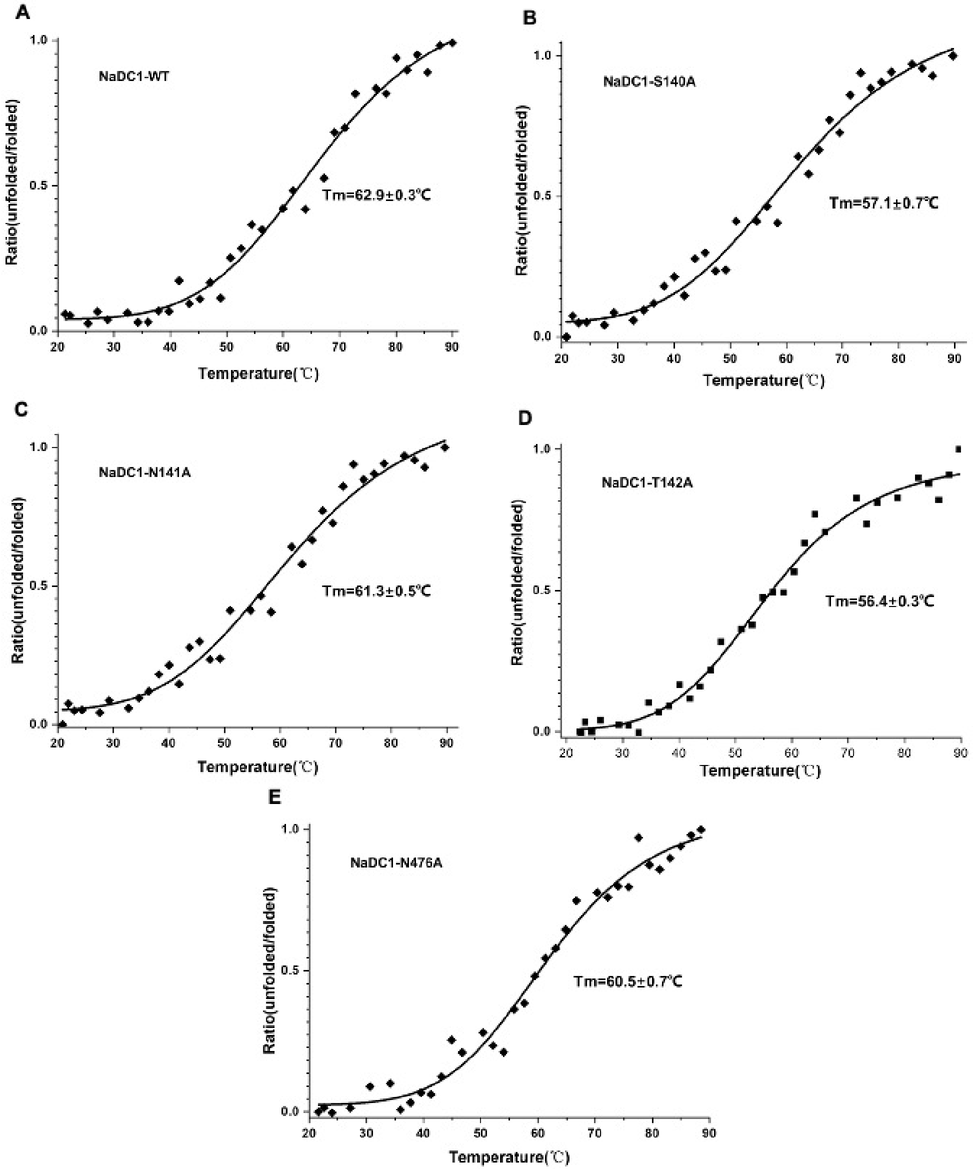

Fig. S12. CD melting curves of NaDC1 and its variants. (**A**) NaDC1-WT. (**B**) NaDC1-S140A. (**C**) NaDC1-N141A. (**D**) NaDC1-T142A. (**E**) NaDC1-N476A. The protein was dissolved at a concentration of 0.75mg/mL in 25 mM Hepes (pH 7.5), 150 mM NaCl, and 0.02% (w/v) Glyco-diosgenin (GDN). Changes in the CD spectrum were recorded at 222 nm as described in Materials and methods. Data are shown for wild-type NaDC1 and S140A/N141A/T142A/N476A variants in the presence of 10 mM sodium citrate. Each curve was repeated independently three times.

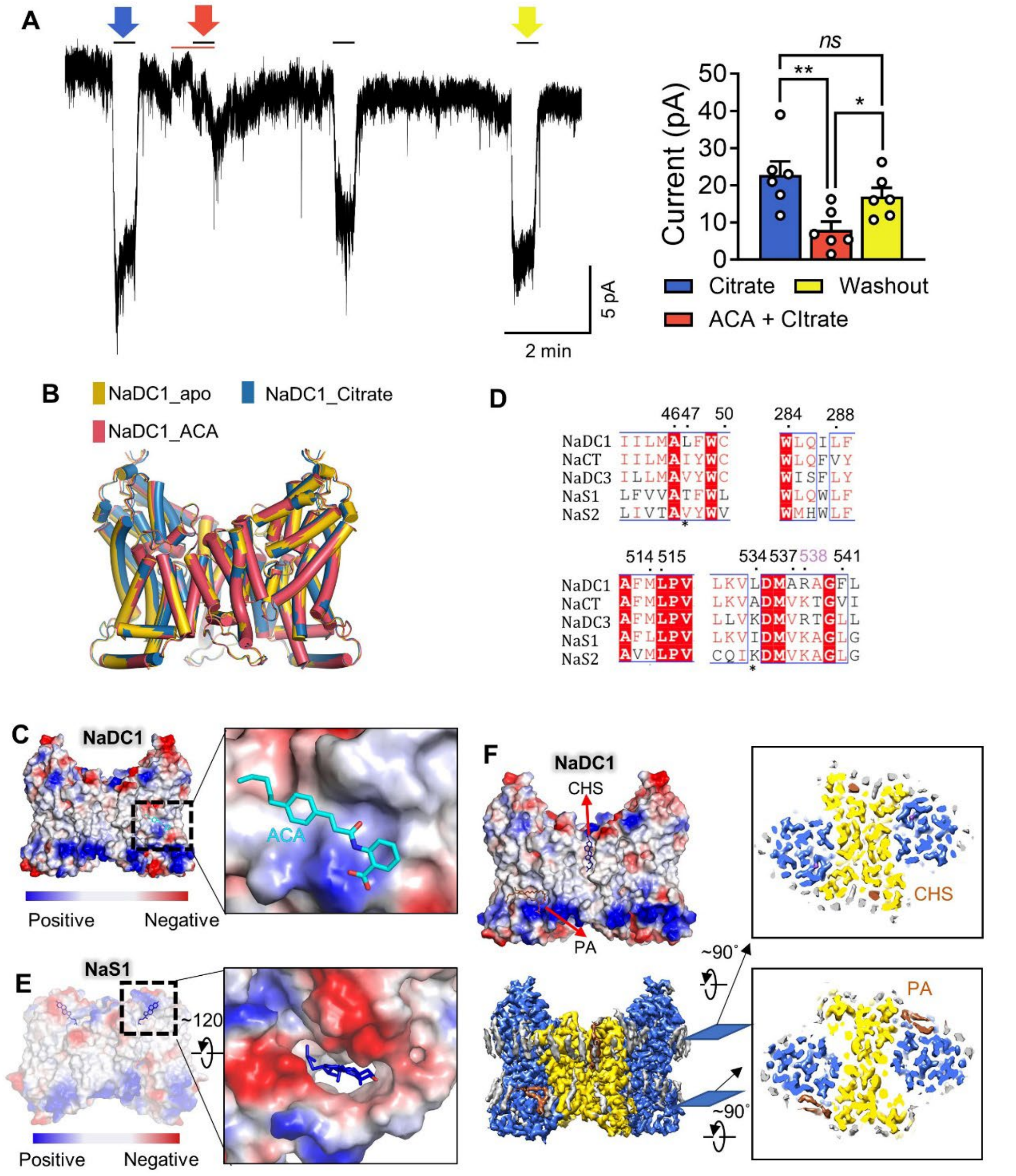

Fig. S13. Lipids and inhibitor binding in NaS1 and NaDC1. (**A**) ACA reversibly inhibit NaDC1 transport activity. Cells were treated with 10 mM Citrate first (show as black bar), followed by co-application of 50 μM ACA and citrate. Citrate was applied again after ACA was washed out. * $p < 0.05$, ** $p < 0.01$ and ns, no significant, calculated by t-test. Data present Mean ± SEM. (**B**) The structural comparison among NaDC1_apo, NaDC1_citrate, and NaDC1_ACA. (**C**) ACA is located in a hydrophobic binding pocket in the peripheral region of NaDC1. The surface is colored according to the surface electronic state. The blue and red are represented positive and negative

charges. The enlarged panel indicates the positive charged patch around the carboxyl group of ACA. (**D**) Sequence alignment analysis of key residues in ACA binding. Major difference includes Leu47 and Leu534. Arg538 conserves in SLC13A3 (NaDC3), and mutates to similar residue of lysine in SLC13A5 (NaCT), SLC13A1 (NaS1) and SLC13A4 (NaS2). All residues numbered after NaDC1. (**E**) Cholesterol-like molecules are imbedded in hydrophobic pockets of NaS1. The surface is colored according to the surface electronic state. Cholesterol-like molecules step in the narrow groove between HPout and TM9c of NaS1. GDN was built considering the overwhelmed addition in protein purification process. GDN is stabilized through hydrophobic interaction. (**F**) Despite phospholipid binding in NaDC1, several rod-like densities can also be detected. CHS was built due to overwhelmed addition in protein purification process. Cross-sections of CHS and phospholipid binding are shown in the panels. Phospholipid-like molecules are attached to the side of HPin, TM4c, TM6b, and TM7 of NaDC1. The branched tail indicates phospholipids. The polyalkylated tail is bound to the hydrophobic pocket of HPin and TM4c. It is further stabilized through electrostatic interaction between polar head of phospholipid and alkaline residues Arg112, His319, and Arg108.

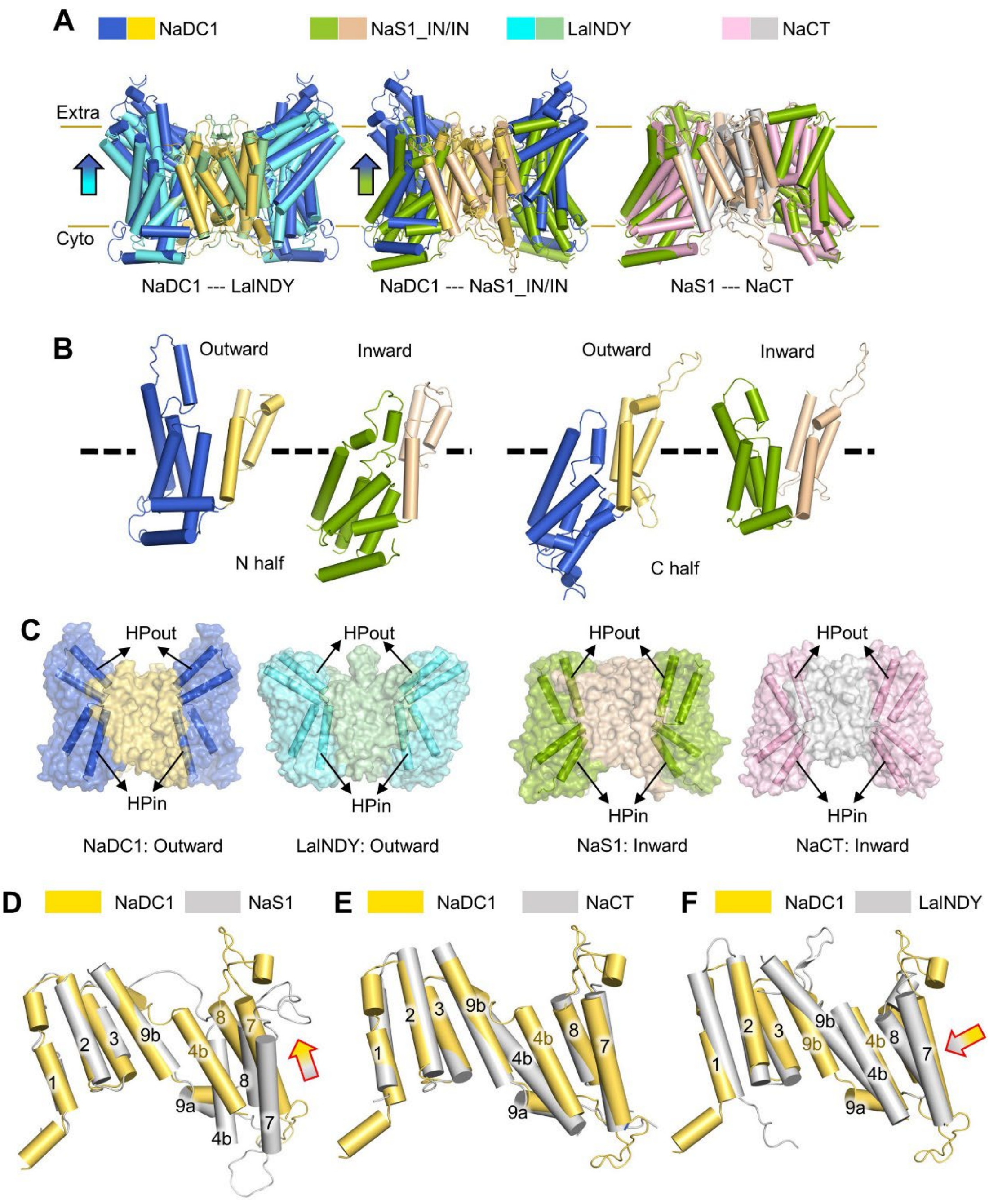

Fig. S14. Conformational difference between SLC13s. (**A**) Left: conformational comparison between NaDC1 and LaINDY (PDB ID: 6WU1). The structure of LaINDY is reported to be in the outward conformation. When the scaffold domains from NaDC1 and LaINDY is aligned, the core domain of NaDC1 moves further towards the extracellular space, indicating a new outward-open conformation. Middle, conformational comparison between NaDC1 and NaS1. The structures are aligned to the scaffold domain, the core domain of NaDC1 moves towards the extracellular space. Right, conformational comparison between NaS1 and NaCT (PDB ID: 7JSK). When

the scaffold domains are aligned, the conformational difference of core domain between NaS1 and NaCT is not obvious. (**B**) The N and C half of NaDC1 and NaS1. The N half structure of NaDC1 (outward) is like C half NaS1 (inward), while the C half structure of NaDC1 is like N half of NaS1. The possible conversion between N and C halfs could be the conformational change during transport process. (**C**) Hairpins conformational comparison. The HPout tilts to horizontal orientation when transporter shift from outward to inward state. The HPin tilts contradictory. (**D**) Comparison between the scaffold domain of NaDC1 and NaS1. TM4b, TM8 and TM7 forms the movement part of the scaffold domain. (**E**) Conformational difference between NaDC1 and NaCT. Only TM4b in the scaffold domain undergo small conformational change. (**F**) Compared with NaDC1, the movement part of the scaffold domain (TM4b, TM8 and TM7) in LalNDY moves towards the paper surface; and TM9b also tils around 15° counterclockwise.

Table S1

Cryo-EM data collection, refinement, and validation statistics of NaS1.

	NaS1_apo_IN/IN	NaS1_apo_IN/OUT	NaS1_sulfate_IN/IN	NaS1_sulfate_IN/OUT
	8W6O	8W6T	8W6H	8W6N
	EMD-37330	EMD-37332	EMD-37323	EMD-37329
**Data collection and Processing**				
Microscope	FEI Titan Krios	FEI Titan Krios	FEI Titan Krios	FEI Titan Krios
Voltage (kV)	300	300	300	300
Camera	K3	K3	K3	K3
Magnification	81000	81000	81000	81000
Pixel size at detector (Å/pixel)	1.087	1.087	1.087	1.087
Total electron exposure (e-/Å^2)	50	50	50	50
Exposure rate (e-/pixel/sec)	23	23	23	23
Number of frames collected during exposure	32	32	32	32
Defocus range (μm)	1.0–2.0	1.0–2.0	1.0–2.0	1.0–2.0
Automation software	AutoEMation	AutoEMation	AutoEMation	AutoEMation
Energy filter slit width (eV)	20	20	20	20
Micrographs collected (no.)	3,321	3,321	5,807	5,807
Micrographs used (no.)	3,042	3,042	5,017	5,017
Total extracted particles (no.)	2,745,237	2,745,237	4,037,817	4,037,817
**Reconstruction**				
Refined particles (no.)	840,751	840,751	712,071	712,071
Final particles (no.)	157,920	169,571	161,063	151,181
Point-group	C2	C1	C2	C1
Resolution (global, Å)	2.9	3	3	3.2
FSC 0.5 (unmasked/masked)	3.6/3.4	3.8/3.6	3.8/3.8	4.2/4.0
FSC 0.143 (unmasked/masked)	3.2/3.1	3.4/3.2	3.4/3.3	3.6/3.5
Resolution range (local, Å)	2.5-3	2.5-3.5	2.7-3.3	2.9-3.5
Map sharpening B-factor (Å^2)	124.4	122.4	144.3	137.8
Map sharpening method	CryoSPARCC	CryoSPARCC	CryoSPARCC	CryoSPARCC
**Model composition**				
Protein (residues)	907	975	923	975
**Model refinement**				
Refinement package	Phenix 1.19	Phenix 1.19	Phenix 1.19	Phenix 1.19
- real or reciprocal space	Real space	Real space	Real space	Real space
-resolution cutoff	0.5	0.5	0.5	0.5
Model-Map scores				
-CC_volume/mask	0.74/0.80	0.73/0.78	0.79/0.83	0.78/0.81
B factors (Å^2)				

Protein residues	26.192	23.845	43.886	41.941
R.m.s deviations from ideal values				
Bonds length (Å)	0.005	0.005	0.009	0.004
Bonds Angle (°)	1.033	0.925	1.059	0.907
**Validation**				
MolProbity score	1.61	1.49	1.68	1.56
CaBLAM outliers (%)	5.5	4.6	4.3	4.1
Clashscore	3.35	2.91	3.43	3.1
Poor rotamers (%)	0.13	0.36	0.76	0.12
C-beta deviations (%)	0	0	0.11	0
EMRinger score	2.7	2.7	2.7	2.7
Ramachandran plot				
Preferred (%)	91.96	93.85	90.25	92.81
Allowed (%)	7.7	6.15	9.52	7.09
Outlier (%)	0.34	0	0.22	0.1

Table S2

Cryo-EM data collection, refinement, and validation statistics of NaDC1.

	NaDC1_citrate	NaDC1_apo	NaDC1_ACA
	8W6C	8W6D	8W6G
	EMD-37320	EMD-37321	EMD-37322
**Data collection and Processing**			
Microscope	FEI Titan Krios	FEI Titan Krios	FEI Titan Krios
Voltage (kV)	300	300	300
Camera	K3	K3	K3
Magnification	81000	81000	81000
Pixel size at detector (Å/pixel)	1.087	1.087	1.087
Total electron exposure (e-/Å^2)	50	50	50
Exposure rate (e-/pixel/sec)	23	23	23
Number of frames collected during exposure	32	32	32
Defocus range (μm)	1.0–2.0	1.0–2.0	1.0–2.0
Automation software	AutoEMation	AutoEMation	AutoEMation
Energy filter slit width (eV)	20	20	20
Micrographs collected (no.)	5,120	5,215	3,721
Micrographs used (no.)	4,284	5,080	2,971
Total extracted particles (no.)	3,771,414	4,789,228	2,268,067
**Reconstruction**			
Refined particles (no.)	1,851,701	1,268,300	916,215
Final particles (no.)	1,369,087	461,481	444,389
Point-group	C2	C2	C2
Resolution (global, Å)	2.7	2.5	3.3
FSC 0.5 (unmasked/masked)	3.3/2.9	3.3/3.0	4.2/3.7
FSC 0.143 (unmasked/masked)	3.0/2.7	2.8/2.5	3.8/3.3
Resolution range (local, Å)	2.6-3	2.4-2.9	3.2-3.6
Map sharpening B-factor (Å^2)	200	100	200
Map sharpening method	Relion	Relion	Relion
**Model composition**			
Protein (residues)	1060	1062	1054
**Model refinement**			

Refinement package	Phenix 1.19	Phenix 1.19	Phenix 1.19
- real or reciprocal space	Real space	Real space	Real space
-resolution cutoff	0.5	0.5	0.5
Model-Map scores			
-CC_volume/mask	0.77/0.83	0.79/0.84	0.82/0.86
B factors ($Å^2$)			
Protein residues	28.16	31.06	62.035
R.m.s deviations from ideal values			
Bonds length (Å)	0.006	0.009	0.007
Bonds Angle (°)	1.005	1.047	0.986
**Validation**			
MolProbity score	1.46	1.22	1.46
CaBLAM outliers (%)	3.4	3.5	3.9
Clashscore	3.34	1.35	2.24
Poor rotamers (%)	0.91	0.23	0
C-beta deviations (%)	0	0	0.2
EMRinger score	2.6	3.2	2.5
Ramachandran plot			
Preferred (%)	95.25	94.86	92.73
Allowed (%)	4.75	4.95	7.27
Outlier (%)	0	0.19	0
